# Multi-institutional Normal Tissue Complication Probability (NTCP) Prediction Model for Mandibular Osteoradionecrosis: Results from the PREDMORN Study

**DOI:** 10.1016/j.ijrobp.2025.12.044

**Published:** 2026-01-08

**Authors:** Laia Humbert-Vidan, Christian R. Hansen, Steven Petit, Carles Muñoz-Montplet, Katrina Hueniken, Abdallah S.R. Mohamed, Deborah P. Saunders, Vinod Patel, Gerda M. Verduijn, Wilma D. Heemsbergen, Arjen van der Schaaf, Max Witjes, Suzanne P.M. de Vette, Mohammad Moharrami, Abdul A. Khan, Jordi Marruecos Querol, Irene Oliveras Cancio, Mike Oliver, Peter Reich, Stacey A. Santi, Andrew G. Pearce, Stephen Y. Lai, Andrew P. King, Ali Hosni, Andrew J. Hope, Erin E. Watson, Johannes A. Langendijk, Jørgen Johansen, Amy C. Moreno, Clifton D. Fuller, Lisanne V. van Dijk, Teresa Guerrero Urbano

**Affiliations:** a Department of Radiation Oncology, The University of Texas MD Anderson Cancer Center, Houston, Texas;; b Department of Medical Physics, Guy’s and St Thomas’ NHS Foundation Trust, London, United Kingdom;; c School of Cancer and Pharmaceutical Sciences, King’s College London, London, United Kingdom;; d Department of Oncology, Odense University Hospital, Odense, Denmark;; e Department of Radiotherapy, Erasmus MC Cancer Institute, University Medical Center Rotterdam, Rotterdam, The Netherlands;; f Department of Medical Physics and Radiation Protection, Catalan Institute of Oncology, Girona, Spain;; g Department of Medical Sciences, University of Girona, Girona, Spain;; h Department of Biostatistics, University Health Network, Toronto, Ontario, Canada;; i Department of Radiation Oncology, Baylor College of Medicine, Houston, Texas;; j Department of Dental Oncology, Health Sciences North, Northern Ontario School of Medicine University, Ontario, Ontario, Canada;; k Department of Oral Surgery, Guy’s and St Thomas’ NHS Foundation Trust, London, United Kingdom;; l Department of Radiation Oncology, University Medical Center Groningen, University of Groningen, Groningen, The Netherlands;; m Faculty of Dentistry, University of Toronto, Toronto, Ontario, Canada;; n Department of Dental Oncology, Princess Margaret Cancer Centre, Toronto, Ontario, Canada;; o Department of Oral and Maxillofacial Surgery, Odense University Hospital, Odense, Denmark;; p Department of Radiation Oncology, Catalan Institute of Oncology, Girona, Spain;; q Department of Medical Physics, Health Sciences North, Sudbury, Ontario, Canada;; r Health Sciences North Research Institute, Sudbury, Ontario, Canada;; s Department of Radiation Oncology, Northern Ontario School of Medicine, Sudbury, Ontario, Canada;; t Department of Head and Neck Surgery, The University of Texas MD Anderson Cancer Center, Houston, Texas;; u School of Biomedical Engineering and Imaging Sciences, King’s College London, London, United Kingdom;; v Department of Radiation Oncology, University of Toronto, Toronto, Ontario, Canada;; w Princess Margaret Cancer Centre, University Health Network, Toronto, Ontario, Canada;; x Department of Clinical Oncology, Guy’s and St Thomas’ NHS Foundation Trust, London, United Kingdom

## Abstract

**Purpose:**

Mandibular osteoradionecrosis (ORN) is a severe late complication affecting patients with head and neck cancer (HNC) treated with radiation therapy (RT) that significantly impacts patients’ quality of life and can require costly interventions. Although radiation dose is a key factor, other clinical and demographic risk factors also influence ORN development. Previous predictive models have primarily been single-institutional, limiting their generalizability. In this first analysis from the PREDMORN Consortium, we have aimed to reproduce existing statistical association and modeling analyses on the largest and most diverse mandibular ORN cohort worldwide to allow comparison with previous studies.

**Methods and Materials:**

This retrospective multi-institutional study included 3928 patients with HNC (622 ORN cases) from 8 institutions. Clinical, demographic, and dosimetric variables were analyzed to develop a prediction model (any grade ORN vs no ORN) using forward stepwise logistic regression with correlation-based variable preselection. The ORN normal tissue complication probability (NTCP) model was developed on 80% of data from 6 institutions, tested on the remaining unseen 20%, and externally validated on a matched cohort (58 patients, 19 ORN cases) and a large population-based cohort (2687 patients, 215 ORN cases).

**Results:**

Key predictors of ORN were D_30%_, V_70Gy_, pre-RT dental extractions, and smoking status. The ORN NTCP model demonstrated very good calibration on the population-based external cohort (Brier score, 0.077; Log Loss, 0.281). Model discrimination improved on a subcohort including oropharyngeal and locally advanced larynx/hypopharynx cancer cases only (AUC from 0.69 to 0.75 and from 0.65 to 0.67 on the matched and the population-based external cohorts, respectively).

**Conclusions:**

The PREDMORN NTCP model is the largest multi-institutional effort to date aimed at predicting ORN risk in patients with HNC using real-world data. The model demonstrated good generalizability when externally validated to a large population-based cohort. Our observations align with current guidelines and corroborate findings from smaller single-institution studies.

## Introduction

Radiation therapy (RT), alone or in combination with chemotherapy and surgery, is the mainstay treatment for head and neck cancer (HNC). Although current external beam RT delivery techniques allow for superior sparing of normal tissues in the head and neck region, treatment-related toxicities are still significant. Mandibular osteoradionecrosis (ORN) is a debilitating late side effect that develops in 4%−15%^1^ of patients with HNC treated with RT. ORN has a detrimental impact on patients’ quality of life,^2^ often necessitating costly clinical interventions.^3^ When the mandible is irradiated, its vascularization is compromised, resulting in hypocellularity of the bone and soft tissues.^4,5^ Eventually, this can develop into necrosis of the bone, which can be diagnosed at different disease stages, ranging from subtle radiographic thinning of the cortical bone to exposed bone or even fracture of the mandible.^6^ Although radiation dose plays an obvious role, understanding the combined contribution of other associated factors for ORN is key to developing effective mitigation strategies. This is particularly important in the context of the observed rise in HPV-associated oropharyngeal cancer (OPC), which has led to a growing population of younger patients with longer overall survival and, therefore, a longer window during which ORN can develop.

Although numerous studies^7–13^ have previously investigated the associations between ORN with demographic, clinical, and dosimetric factors, most models have been developed and validated using only single-institutional cohorts. Comparisons between studies is not straightforward because of the large diversity of cohorts, treatment techniques, ORN classification systems, and inclusion criteria considered. Moreover, the clinical adoption of validated and generalizable conclusions is hampered by the limited size of these data sets, driven by the naturally low prevalence of ORN. For example, a normal tissue complication probability (NTCP) model for mandibular ORN was developed by van Dijk et al,^7^ using a large single-institution cohort, based on dose-volume metrics and clinical and demographic variables. Although this model holds significant clinical value for the institution in which it was developed, its generalizability to other settings may be limited because of potential differences in treatment and patient characteristics.

To address these limitations, the PREDiction models for Mandibular OsteoRadioNecrosis in head and neck cancer (PREDMORN) Consortium^14^ was created, currently consisting of 8 European and North American institutions: Guy’s and St Thomas’ NHS Foundation Trust (GSTT, United Kingdom), The University of Texas MD Anderson Cancer Center (MD Anderson, United States), Odense University Hospital (OUH, Denmark), University Medical Center Groningen (UMCG, Netherlands), Erasmus Medical Center (EMC, Netherlands), Catalan Institute of Oncology Girona (ICO, Spain), Health Sciences North Research Institute (HSNRI, Canada) and Princess Margaret Cancer Center (PMCC, Canada).

In this study, the PREDMORN Consortium aimed to (1) curate the largest and most diverse ORN real-world data set ever considered worldwide; (2) identify independent dosimetric and clinical predictors of ORN; and (3) develop, test, and externally validate an NTCP prediction model for ORN.

## Methods and Materials

### Study design and patient selection

Following the required ethics approvals at each institution (REC reference 18/NW/0297, IRAS project ID: 231443 (Guy’s and St Thomas’ NHS Foundation Trust), RCR-03–800 (The University of Texas MD Anderson Cancer Center); EMC17404 (Erasmus Medical Center); NCT02435576 (University Medical Center Groningen); approval by The Danish Data Protection Agency [Jr. 16/29136] and The Danish Patient Safety Authority [Jr. 3-3013-1798/1/] (Odense University Hospital); CEIC approval date 22/06/2015 (Catalan Institute of Oncology); REB number 23–038 (Health Sciences North Research Institute); approval number 17–5871.19 (Princess Margaret Cancer Center)), subjects with mandibular ORN were retrospectively selected from their entire HNC population considering the inclusion/exclusion criteria described in the study protocol^14^ (Table A1). Initially, the study design was such that control subjects were selected using an approximate 2:1 matched control-to-case approach, anchored to the available ORN cases at each institution, as not all participating centers were able to retrieve data from their entire head and neck cancer population. This approach was followed for data sets from 7 of the 8 participating institutions. Specifically, for each site, the local ORN cases were first identified and their distribution analyzed with respect to primary tumor site and treatment year, as described in the study protocol.^14^ Controls were then selected to match these distributions as closely as possible. The following primary tumor site groups were considered: OPC, oral cavity cancer (OCC), larynx/hypopharynx cancer, and others, the latter including cases of salivary gland cancer, nasopharynx cancer, and unknown primary tumor sites. At a later stage (after the model had been developed, tested, and validated using the initial cohort), an eighth institution joined the consortium with a large population-based data set, which was used to carry out an additional external validation. Figure A1 provides a visual representation of the study design described here. Actual ORN prevalence rates ranged from 5.1% to 13.7% across all participating institutions. However, the ORN incidence in the initial cohort (ie, data from 7 of 8 institutions) of this study does not represent the actual HNC population ORN rates.

### Data

#### Clinical data

Clinical data were collected by each institution using a standardized data collection template with predefined variable codes (Table A2). Because of the retrospective study design, some centers encountered missing data. Variables with a percentage of missing data >10% over the entire cohort were excluded from the analysis. Patients with missing values in the included variables were excluded. Based on the calculated percentages of missing data (Tables B1 and B2), the following subset of clinical and demographic variables were selected for statistical modeling: sex (binary, male vs female), age (continuous), mandible volume in cm^3^ (continuous), smoking status (binary, current vs never/former), pre-RT dental extractions (binary, yes vs no), chemotherapy (binary, yes vs no), PORT (binary, PORT vs primary RT), RT technique (binary, IMRT vs VMAT), and fraction size (> vs ≤2 Gy/fraction). Although well recorded, T and N stage were not included as covariates, as they are clinical determinants of radiation therapy (RT) dose prescription and the use of concurrent chemotherapy and were therefore considered indirectly represented through dosimetric and treatment-related variables. Primary tumor site was also not included as a covariate as this was the variable used for cohort matching.

#### Dosimetric data

Segmentation of the mandible was performed locally within each institution on the treatment planning computed tomography images. Although the segmentation method varied across institutions, ranging from manual delineation to atlas-based auto-segmentation software (eg, ADMIRE, Elekta), the protocol uniformly defined the structure to include the condyles and exclude the teeth. Dosimetric data for the mandible structure were extracted from the treatment planning systems as the cumulative DVH in absolute dose (Gy) and relative volume (%), and the following dosimetric variables were calculated: D_2%_ (where D_x%_ corresponds to the dose received by X% of the mandible volume), D_5%_-D_95%_ in 5% steps, D_98%_, and V_5Gy_-V_70Gy_ (where V_XGy_ is the volume of the mandible that receives at least X Gy) in 5 Gy steps. No fractionation correction was performed; instead, physical dose was used and the prescribed fraction size was considered as a variable to account for the potential effect of differences in fractionation schemes.

#### Clinical endpoint

Because of the variability between centers in ORN grading system (eg, Notani,^15^ Tsai,^16^ see Table B3) and ORN intervention strategies, a uniform classification system across all participating centers could not be realized, and where feasible, retrospective reclassification of ORN cases into Notani stages was performed. However, direct translation from the Tsai system to the Notani system was not possible at certain centers, as the 2 systems differ fundamentally: Notani’s system is based on anatomical boundaries (ORN confined vs beyond the alveolar bone), whereas the Tsai system grades the severity of the bone exposure based on the required clinical intervention. To enable consistent outcome definition across the consortium, the ORN endpoint was dichotomized as “no ORN” versus “any stage of ORN,” regardless of the original classification system. This binary endpoint was based on clinical and/or radiological diagnosis according to each center’s standard protocols, allowing inclusion of all contributing institutions in the analysis while maintaining a harmonized definition of ORN occurrence.

### Statistical analyses and NTCP model development

Data from six institutions were used to develop the described model. The model development data set was partitioned into training (80%), and independent test (20%) sets using a stratified split (ie, equal ratio of ORN vs control subjects in both sets). This split was done without matching the distribution of any of the covariates considered for analysis. Bootstrapping was performed to assess the robustness of variable selection and model performance. The 2 external validation cohorts considered originated from 2 institutions that joined the consortium only after model development had been completed using data from the initial participating sites. This sequencing was intentional and ensured that the external data sets remained entirely independent of the training and testing process, thereby allowing for an unbiased and robust external evaluation of model performance. Because of timing limitations in addressing legal constrains with regard to data sharing, a data sharing agreement was not in place with the eighth institution and data aggregation with the other data sets was not possible in this study. Thus, their population-based data set was primarily used for validation of the model and other institution-specific associated analyses using a federated learning approach (ie, data analyses scripts and final results were shared instead of raw data).

For explorative purposes, univariable logistic regression analysis was performed to assess the individual statistical relevance of both the clinical and dosimetric variables. Variables were ranked according to their level of significance (*P*value) with regard to their association with ORN. For the NTCP model development, a multivariable forward stepwise logistic regression with a prior variable preselection approach was followed. First, redundant variables were excluded through a preselection procedure to avoid multicollinearity: if the correlation between pairs of variables was high (Spearman correlation coefficient > 0.8) then the variable of that pair with the lowest univariable association to ORN (highest *P*value) was omitted. Second, these preselected variables were subjected to a forward stepwise variable selection process, adding one variable to the model in every step (ie, model with 1 variable in step 1, then added one more in each subsequent step). Candidate variables (those not yet selected) were ranked for each step based on the Akaike Information Criterion (AIC) values, where the variable corresponding to the lowest AIC was selected. A second criteria to add the variable to the model was a significant improvement according to a likelihood-ratio test with a threshold *P*value of .05. The variable selection procedure was repeated 5000 times on bootstrapped samples of the training data sets.

The final model performance was evaluated using several metrics. Model fit was assessed by the AIC and Bayesian Information Criterion. Discriminative ability was quantified using the receiver operating characteristic (ROC) curve with the area under the curve (AUC) and its 95% CI. The Nagelkerke *R*^2^ measured the explained variance, whereas calibration was evaluated through calibration plots, the Brier score, and Log Loss.

### Subcohort analyses

Radiation dose distributions and treatment protocols are typically tumor site-specific. The distribution of dose across the different subsites varies, and, in addition, the standard primary treatment for locally advanced OCC is surgery followed by postoperative RT (PORT) sometimes combined with concurrent chemotherapy. In contrast, other subsites are predominately treated with primary RT, often combined with concurrent chemotherapy. To account for this, the following subcohort analyses were performed for (1) a subset of subjects including OPC and locally advanced (T3/T4) larynx/hypopharynx cancer cases; (2) a subset including OCC cases only; (3) a subset including subjects treated with primary RT; and (4) a subset of subjects treated with PORT.

## Results

### Data

The initial cohort consisted of 3950 patients with HNC, of which 630 were ORN cases. Table 1 provides a summary of the clinical and demographic data (institution and data set-specific cohorts are described in additional tables in Supplement B). After the exclusion of missing data, a final data set of 3928 subjects (622 ORN cases) was used for analysis. The training data set consisted of 948 subjects (309 ORN cases), with a median (IQR) follow-up time of 4.7 (3.2–6.1) and 4.4 (2.7–5.8) years for the ORN and control groups, respectively. The test data set consisted of 236 subjects (80 ORN cases), with a median (IQR) follow-up time of 5.0 (3.2–6.0) and 4.1 (3.0–5.5) years for the control and ORN groups, respectively. The first (matched) external validation cohort consisted of 57 subjects (18 ORN cases), with a median follow-up time of 3.7 (2.4–4.7) and 4.8 (1.7–6.1) years for the control and ORN groups, respectively. The second (population-based) external validation cohort consisted of 2687 subjects (215 ORN cases), with a median (IQR) follow-up time of 4.8 (1.9–6.3) and 5.9 (4.1–8.2) years for the control and ORN groups, respectively. Significant variation was observed in most clinical and treatment-related variables across the train, test, and external validation cohorts (Table B1), as well as among the different participating centers (Table B2).

#### Clinical data

Across the matched cohorts (institutions A-G), the dominating primary tumor site was OPC (63.0%), followed by OCC (28.5%), larynx/hypopharynx (5.0%), and other sites (3.6%). For the population-based cohort (institution H), these percentages were 35.9%, 18.1%, 10.8%, and 35.2%, respectively. Although tobacco smoking status was included as a variable for statistical analyses with observed statistical association to ORN (*P* = .002) at univariable analysis (see Table C1), the amount of tobacco (tobacco pack years) was not uniformly well recorded. However, based on a subset of 368 controls and 185 ORN subjects across the matched cohorts, the median (IQR) of pack years for ORN exceeded that of the controls: 10.0 (0.0–34.0) vs 3.0 (0.0–30.5), although this difference was not statistically significant (*P* = .094). In the population-based cohort, the median was also larger in the ORN group: 20.0 (0.0–40.0) vs 10.0 (0.0–30.0). Dental extractions pre-RT were performed in a higher percentage within the ORN group (49.9% vs 42.7%) with a statistically significant association with ORN at univariable analysis (*P* = .026). This was also the case for the population-based cohort, where 38.6% and 34.7% of patients had extractions in the ORN and control groups, respectively. Although information on the elapsed time between dental extractions and the start of RT was largely missing, on a subset of 174 controls and 52 ORN cases with available data across the matched cohorts, the median (IQR) time was shorter for ORN cases: 20.0 days (14.0–31.5) vs 28.0 days (20.0–47.0) and this difference was found statistically significant (*P* = .002). Chemotherapy was more common in ORN cases (68.7% vs 64.2%) and was statistically associated with this endpoint (*P* = .042). This was also the case for the population-based cohort, with percentages of 47.9% and 42.8% for the ORN and control groups, respectively. Xerostomia at 1-year post-RT was recorded for 455 (36.0%) subjects (305 controls and 150 ORN), and a significantly (*P* = .015) higher percentage of grade ≥2 xerostomia was observed for the control group (22.1% vs 17.8%). Mandible volume varied largely across institutions (Fig. D1) and was significantly associated with ORN at univariable analysis (*P* = .013) with ORN cases exhibiting larger volumes, 72.5 cc (60.6–87.6) vs 69.6 (56.8–83.1). Mandible volume, however, was significantly associated to PORT status (*t* test *P*value < .001), with smaller mandible volumes observed in patients who underwent surgery prior RT (ie, PORT positive status) (median 64.4 cc, IQR, 52.3–75.3) compared with patients receiving RT as their primary treatment (73.1 cc, 60.6–87.0) (see Fig. D2).

#### Dosimetric data

**Fig. 1.**
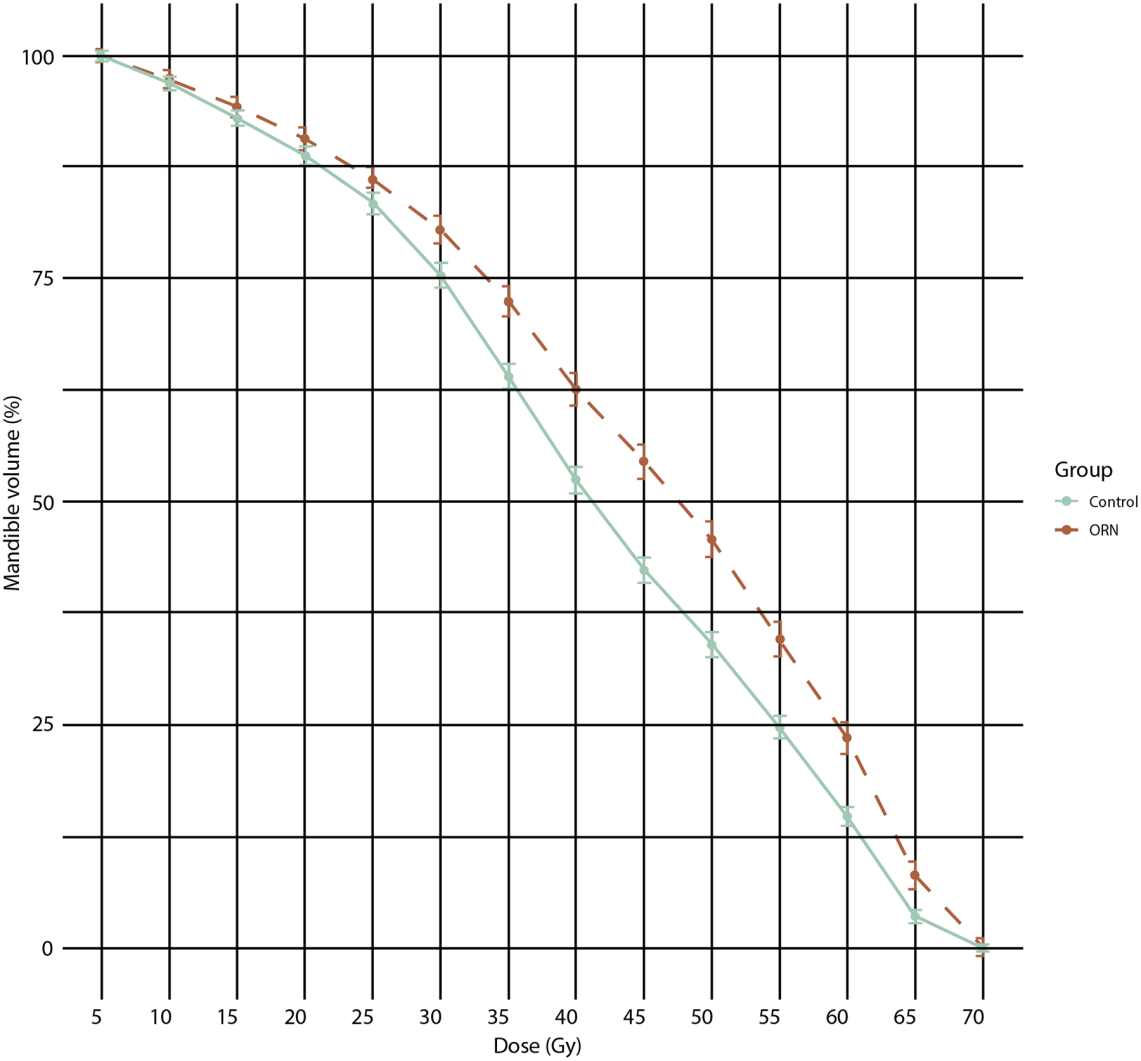
Median cumulative DVH plots for the ORN and control groups of the PREDMORN cohort (matched data sets). The medians were calculated for each DVH metric across the ORN and control groups. Error bars correspond to the 95% confidence intervals of the medians estimated using the binomial method, which identifies the lower and upper ranks corresponding to the 95% confidence bounds based on the size and distribution of the data. Additional institution-specific DVH plots (Fig. E1) and DVH plots for the OPC/advanced larynx cancer and OCC subcohorts (Fig. E2) are provided in Supplement E. *Abbreviations:* DVH = dose-volume histogram; OCC = oral cavity cancer; OPC = oropharyngeal cancer; ORN = osteoradionecrosis.

All dosimetric variables showed significant variation across the participating centers (ANOVA *P* < .001). The median cumulative mandibular DVH was higher for the ORN group than for the controls, as shown in Figure 1; this was also true for individual institution-specific DVH data (see Fig. E1). In particular, for the matched cohorts, the median (IQR) V_50Gy_ to the mandible was 45.8% (31.8–61.6) and 34.1% (23.3–50.2) for the ORN and control groups, respectively (*P* < .0001) and the median D_2%_ to the mandible was 67.6 Gy (63.4–71.3) and 66.3 Gy (62.2–69.4) for the ORN and control groups, respectively (*P* < .0001). For the population-based cohort, the median (IQR) V_50Gy_ to the mandible was 44.5% (30.7–61.8) and 32.5% (20.0–49.5) for the ORN and control groups, respectively, and the median D_2%_ to the mandible was 68.2 Gy (62.7–70.6) and 65.8 Gy (60.8–69.0) for the ORN and control groups, respectively. On univariable analysis on the matched cohorts, most DVH metrics were significantly related to ORN (*P* < .05), except for V_5Gy,_ V_10Gy_, V_15Gy_, D_90%_, D_95%_, and D_98%_; a smaller set of significant DVH metrics was observed on the OCC subcohort (see Table C1). Parotid gland doses were available in 21.7% of subjects (196 controls and 78 ORN cases), with mean doses for the ORN group marginally higher than for the control group, with a median (IQR) of 27.8 Gy (20.6–34.1) vs 26.3 (18.6–33.2). Figure E3 describes the distribution of fraction size across the train, test, and external subsets. Figure E4 provides the corresponding distributions of fractionation schemes.

#### Clinical endpoint

Of all 622 ORN cases, 162 (26.0%) were advanced grade ORN (either Notani grade III or Tsai grade 4). The observed median (IQR) time from the end of RT to ORN diagnosis was 1.2 years (0.6–2.3) and 1.4 years (0.6–3.1) for the matched data sets and population-based data set, respectively, with a range across centers of 0.6 to 7.4 years (see Fig. F1). A statistically significant difference in time to ORN curves was observed between patients with and without pre-RT dental extractions (*P* = .002) and between patients with and without a history of smoking (*P* = .012). Patients who underwent pre-RT dental extractions or with a positive smoking status (ie, current smoker) showed a higher probability of developing ORN earlier, with an increase of up to 18.7% and 13.2% at 0.8 and 3.2 years, respectively (see Figs. F2a and b, respectively).

### Variable preselection

All 8 clinical variables considered for multivariable statistical analysis—Pre-RT dental extractions, smoking status, chemotherapy, fraction size, sex, RT technique, PORT, and age—were preselected based on their low collinearity, as determined by the Spearman correlation test (Fig. G1 and Table G1). This outcome was anticipated, given that the proposed set of clinical variables was intentionally designed to capture potential clinical risk factors without redundancy. Dose-volume variables, however, are inherently correlated because they originate from a single source (DVH). Consequently, during the multicollinearity reduction process, only a subset of 5 dose-volume variables (D_10%_, D_30%_, D_60%_, V_25Gy_, and V_70Gy_) was selected for further analysis.

### Multivariable ORN NTCP model

**Fig. 2.**
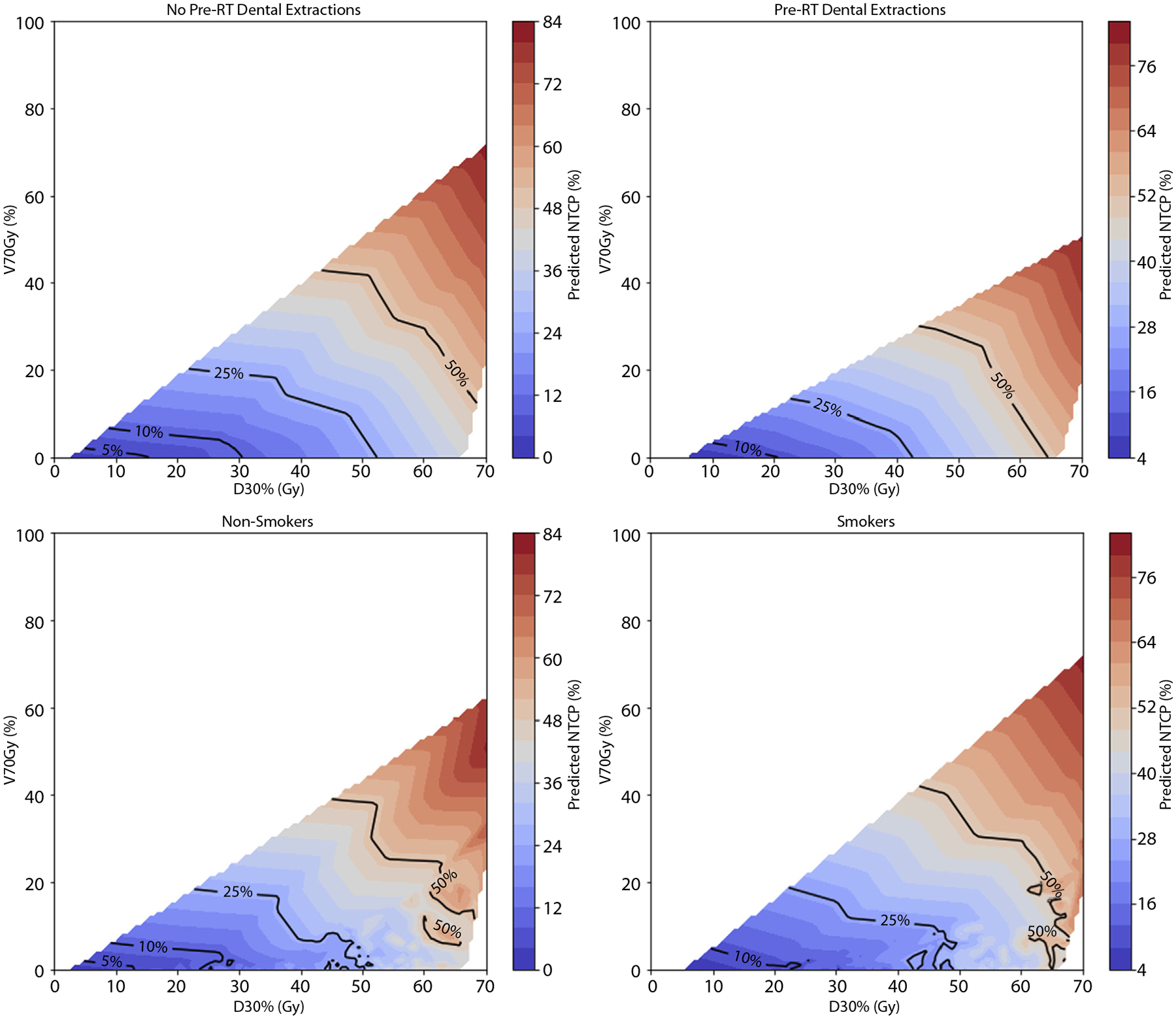
Normal tissue complication probability (NTCP) contour plots for mandibular osteoradionecrosis (ORN) risk as a function of D_30%_ and V_70Gy_, stratified by (a) pre-RT dental extractions and (b) smoking status. The NTCP values were interpolated across the dose range for visualization. Abbreviation: RT, radiation therapy.

The forward stepwise selection process (Table G2) identified the following variables as the best set of predictors for ORN in the following order of importance: D_30%_, pre-RT dental extraction, V_70Gy_, and smoking status. These variables remained the most frequently selected in the bootstrapped analyses (Fig. G2). The distribution of V_70Gy_ (Fig. G3) revealed that most patients had values near 0%. Although V_70Gy_ reached statistical significance, the observed effect size was negligible (Cliff’s d = −0.13), suggesting that its contribution to the model may be driven by statistical rather than clinically meaningful differences. Nonetheless, D_30%_ and V_70Gy_ were only weakly correlated (Spearman’s r = 0.32, *P* < 2.2 × 10^−16^, Fig. G4). Positive regression coefficients were obtained for all model parameters (Table 2), indicating that an increase in these risk factors resulted in a higher mandibular ORN risk. In particular, D_30%_ values ≥ 45 Gy and V_70Gy_ ≥ 10% were associated with a 3-fold increase in predicted ORN risk relative to patients with lower values for both metrics (50.6% vs 16.8%). Similarly, the presence of pre-RT dental extractions (Fig. 2a) and smoking status (Fig. 2b) amplified predicted risk in combination with these dosimetric factors (Fig. 2).

### ORN NTCP model performance

**Fig. 3.**
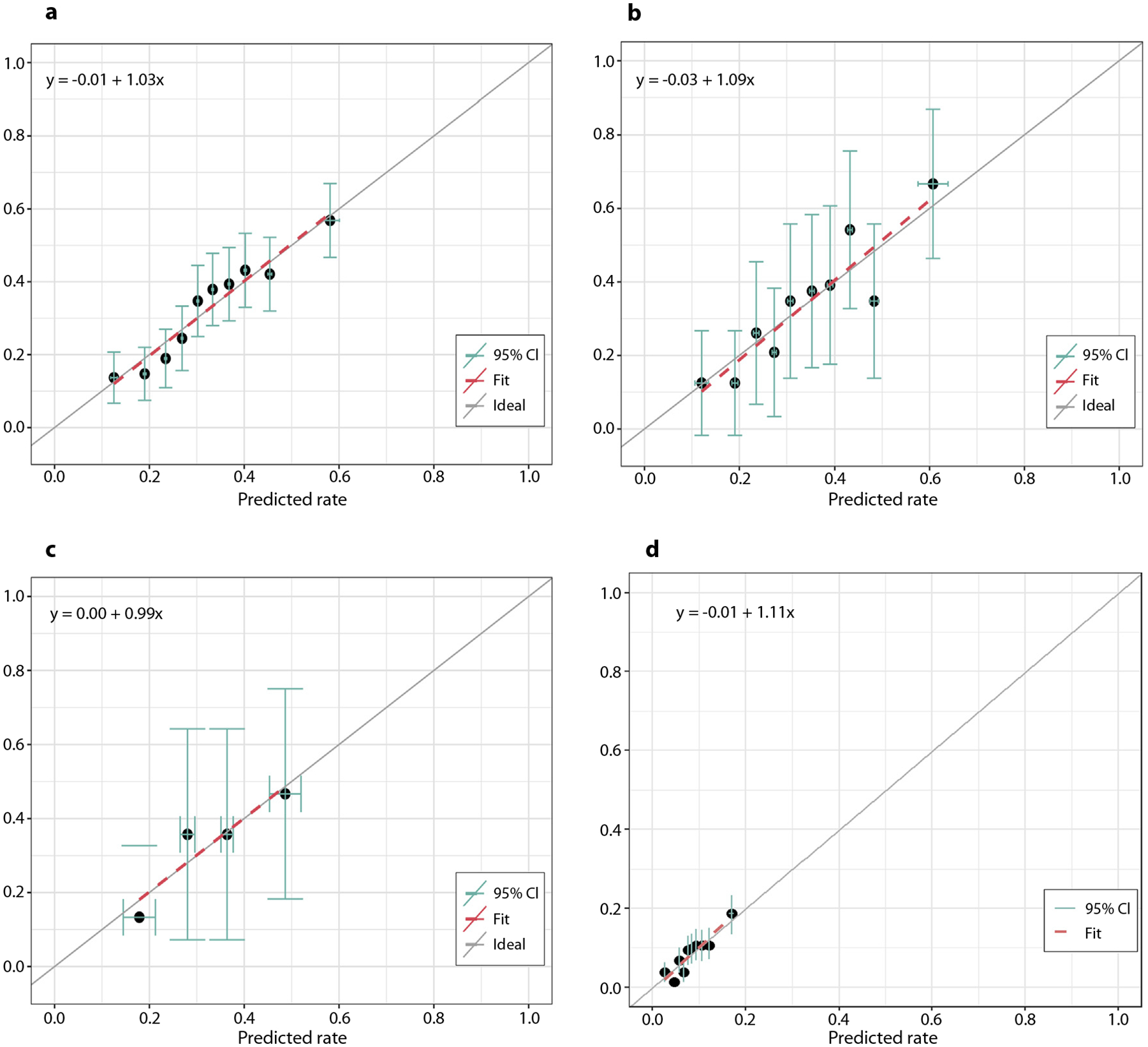
Calibration curves when testing the ORN NTCP (normal tissue complication probability) model on the (a) train, (b) test, (c) first external validation, and (d) second external validation data sets. The number of bins in the calibration plots was adjusted in each plot based on data set size for each cohort/subcohort to optimize representation of the data (ie, 10 bins for the train, test, and second external validation calibration plots, 4 bins for the first external validation plot). Abbreviation: ORN, osteoradionecrosis.

The ORN NTCP model demonstrated moderate overall performance with AUCs of 0.67 (95% CI, 0.64–0.71), 0.69 (0.62–0.76), 0.69 (0.53–0.84), and 0.65 (0.61–0.69) on the train, test and 2 external validation data sets, respectively (Table 3). Calibration plots demonstrate good model calibration in both the train (Fig. 3a), test (Fig. 3b), and 2 external validation cohorts (Fig. 3c, d), as the predicted and observed rates matched the identity line closely (intercept range, −0.03–0.00; slope range, 0.99–1.11).

### Subcohort analyses

When applied to the OPC/locally advanced (T3/T4) larynx/hypopharynx cancer subcohort, the model’s performance improved (see Table H1 and Fig. H1), with increasing ROC AUC (from 0.69 to 0.75 for both the test and the first external validation data sets and from 0.65 to 0.67 for the second external validation), and higher Nagelkerke *R*^2^ values, indicating better discrimination and fit. The Brier scores and Log Loss also improved, reflecting enhanced accuracy in this subcohort. Similarly, the model’s performance was higher when tested on the primary RT subcohort (see Table H2 and Fig. H2). Conversely, the model’s performance decreased when tested on the OCC or PORT subcohorts.

## Discussion

In this first analysis performed by the PREDMORN consortium, we have aimed to reproduce existing statistical association and modeling analyses on the largest and most diverse multi-institutional mandibular ORN cohort worldwide to allow comparison with previous studies. As such, we have developed, tested, and externally validated an NTCP model for ORN.

On a cohort of 3928 subjects (622 ORN cases), the multivariable ORN NTCP model identified key risk factors as D_30%_ (the dose received by 30% of the mandible volume), pre-RT dental extractions, V_70Gy_ (the mandible volume receiving ≥70 Gy), and smoking status.

Our model showed a moderate predictive performance (AUC of 0.69 for testing and the first external validation, AUC of 0.65 for the second external validation), but improved accuracy in OPC and locally advanced (T3/T4) larynx/hypopharynx (AUC 0.75, 0.75, and 0.67) and primary RT (AUC 0.70, 0.72, and 0.67) subcohorts. The model was well-calibrated, with good agreement between predicted and observed risks across all validation data sets, including in the population-based cohort (Log Loss, 0.281).

Higher radiation doses are a known risk factor for ORN and the 2024 ISOO-MASCC-ASCO guidelines^17^ state that patients receiving radiation doses to the mandible higher than 50 Gy should be considered at risk of developing ORN. A recent large-scale single-institution study^18^ suggested a D_10cc_ threshold of 59.2 Gy as the most discriminative between ORN and non-ORN subjects. Previous studies, however, have also reported differences between the ORN and control groups at low doses^10^ and suggested microvasculature collapse in the mandible at doses around 30 Gy.^19^ In van Dijk et al,^7^ D_30%_ was also selected in their NTCP models, with thresholds of 35 and 42 Gy for a <5% risk of ORN in patients with and without predental extractions, respectively. Our model identified slightly lower thresholds for the same risk level, with D_30%_ values <10 Gy for smokers or dental extractions and <20 Gy otherwise for the matched cohort. In the population-based cohort, the corresponding thresholds had values of ≤26 Gy and 43 Gy, respectively. These differences in D_30%_ threshold values could be influenced by the differing ORN prevalence rates. Notably, although our model was developed on a 2:1 matched cohort, with an artificially elevated ORN incidence rate of 32.9%, its calibration was successfully validated in a large population cohort with a slightly lower prevalence rate (8.0%) to that used in van Dijk et al^7^ (13.7%).

Although both dosimetric variables D_30%_ and V_70Gy_ were retained as predictors in the final NTCP model, the clinical interpretation of V_70Gy_ warrants caution, given its distribution and limited effect size. Nonetheless, the weak correlation between the 2 parameters suggests that they reflect distinct aspects of the dose distribution, supporting the inclusion of both in the model. For the institutions with matched data sets, the median D_30%_ (IQR) across the ORN and control groups was 57.4 Gy (51.4–61.9) and 52.5 Gy (45.1–71.9), respectively. Similar values were observed for the population-based data set, with median values of 56.6 (50.4–60.8) and 51.6 (42.8–57.6) for the ORN and control groups, respectively.

The risk of ORN escalates with dental extractions or surgery, as the compromised vascularization in an irradiated mandible impairs the mandible’s healing capacity after such procedures, where the time between dental extraction and the start of RT to allow for the healing process plays a crucial role.^20^ The ISOO-MASCC-ASCO guidelines^17^ recommend a “2-week healing period between the time of dental extraction and the start of radiation therapy”; most participating PREDMORN institutions met this recommendation, with slightly overall shorter times observed in ORN cases (20.0 vs 28.0 days). In line with previous work,^9^ dental extraction pre-RT was observed to negatively impact the longitudinal risk of developing ORN. Further investigation should focus on a more complete data set of time from extractions to RT start and the association between extraction site and ORN localization.

As highlighted in previous studies,^19,21,22^ there is an increased vulnerability to ORN in the HPV-associated OPC group of patients, who are generally younger, with better dental status, and more often without the lifestyle factors traditionally associated with ORN such as smoking,^23^ challenging the stigma that patients with HNC have neglected and poor dentition.^24^ However, for the matched data sets, we found similar smoking rates (*P*= .91) in the OPC sub-group (28.3% and 42.4% for the control and ORN groups, respectively) compared to other primary sites (30.5% and 44.6% for the control and ORN groups, respectively).

Patients with HNC, mostly OCC cases, may undergo pre-RT mandibular surgery, such as rim resection or hemimandibulectomy. For the matched data sets in the PREDMORN cohort, 81.2% of OCC cases were treated with PORT, where the whole remaining mandible is typically irradiated to a more homogeneous dose compared to OPC or locally advanced larynx/hypopharynx cases. This results in lower discriminative potential for the DVH variables, especially in lower and intermediate doses (as shown in Fig. E2b), which could have contributed to a worse model performance for the OCC and PORT subcohorts.

A recent study by Verduijn et al^25^ also observed an association between mandibular volume and ORN and speculated that this could be related to the dentition status of the patient. In the matched cohorts of our study, mandible volume was found to be statistically associated with ORN at univariable analysis for the OCC subcohort (*P* = .024) but not for the OPC/locally advanced larynx/hypopharynx subcohort (*P* = .079). On the other hand, mandible volume was found to be statistically associated with PORT status, with smaller mandible volumes observed in patients treated with PORT (median 64.4 cc; IQR: 52.3–75.3) versus primary RT (median 73.1 cc; IQR: 60.6–87.0) (see Fig. D2). Further investigation is required concerning this less explored risk factor. The irradiation of a partially resected mandible may contribute to the risk of ORN because of the combined effects of surgery and radiation compromising its vascularization. Building on previous studies^5,26,27^ that have characterized imaging parameters linked to vascular damage in the irradiated mandible, future research should leverage imaging data to further elucidate the interplay between pre-RT surgery, mandibular volume, and ORN incidence.

This study has some limitations. A matched control-case approach based on primary tumor site was followed as population-based consecutive data sets were not available from all participating institutions. This potentially introduced bias in the representation of the tumor sites within the matched cohort both across participating institutions and when compared with previous studies (eg, the proportion of larynx cases we considered was smaller than in the population-based cohort considered in the study by van Dijk et al^7^). Differences in case mix and ORN incidence rate (ie, natural ORN incidence in a consecutive data set compared to that in a 2:1 matched cohort) could account for the superiority of their model’s discrimination performance. Nonetheless, our model demonstrated good calibration when externally validated on a large, population-based cohort, reinforcing its generalizability beyond the matched development data set and its relevance to broader clinical populations.

Another limitation of this study is the variability in mandible segmentation methods across participating institutions. As the analysis largely relied on retrospectively collected data, delineations were based on each institution’s local practice rather than being standardized from the outset. Although all segmentations followed a common structural definition (including the condyles and excluding the teeth), methodological differences may have introduced interinstitutional variability in the dosimetric analysis. Planned work involving image-based modeling will utilize raw DICOM dose distributions and apply a standardized, centralized segmentation protocol to ensure better consistency and reproducibility across data sets.

Moreover, raw DVH data were not available from all participating institutions; therefore, only physical dose metrics derived from precalculated DVH summaries could be used in the analysis. As a result, since the dose distribution is not homogeneous within the mandible, voxelwise fractionation correction (eg, EQD2 transformation) could not be applied. To partially account for interinstitutional variability in fractionation schemes, the prescribed fraction size was included as a covariate in the model. Although this approach provides some adjustment, it does not fully capture the biological equivalence of different dose-fractionation regimens. This limitation is particularly relevant for high-dose-volume metrics such as V_70Gy_, where even small changes in fraction size can shift biologically equivalent doses above or below the 70-Gy physical threshold, potentially distorting its relationship with clinical outcomes. However, we would like to note that the effect size of V_70Gy_ was very small in our model, suggesting that its inclusion had limited clinical impact. On the other hand, the large majority of patients in our cohort were treated with 2.0 Gy per fraction, for whom EQD2 and physical metrics are essentially identical. Additionally, we compared our results with those obtained from an approximation of EQD2 conversion applied at the DVH-metric level and showed minimal model performance differences (see tables I1 and I2 and figures I1–I3).

The choice of dose reporting formalism—whether D_w,w_, D_w,m_, or D_m,m_—varied across institutions, where D_x,y_ represents the dose to medium *x* (*w* for water or *m* for other medium) calculated using the energy deposition in medium *y*. This variability likely introduced a degree of systematic uncertainty in the DVH values, particularly in high-density regions such as bone or metal implants.^28,29^ As this study was conducted retrospectively, dose recalculation was not feasible; however, we recommend establishing a standardized dose reporting framework for future studies to ensure consistency and comparability across institutions.

Acknowledging the different stages or grades of ORN is highly relevant as the clinical intervention required will often depend on the severity of ORN, with more severe ORN, involving bone fracture requiring surgical intervention. Because of the lack of consensus in the classification system for ORN, in this study, we dichotomized the endpoint (ORN vs no ORN) rather than being able to consider separate stages. A new staging system, ClinRad, has been recently proposed by Watson et al^6^ that reportedly outperforms existing systems and has been endorsed for prospective use by ISOO-MASCC-ASCO guidelines^17^ to standardize reporting on ORN. A key limitation of modeling ORN as a binary endpoint is the potential for bias introduced by differential follow-up duration. Patients who died early may have been misclassified as not having developed ORN, simply because of insufficient time at risk. This limitation is especially relevant, given that ORN can develop several months or even years after RT. Although a binary endpoint aligns with standard practices in NTCP modeling, it might not capture the temporal dynamics of toxicity development. Ongoing work within our group is addressing this gap by applying time-to-event analysis techniques to model time to ORN onset and its progression.

Existing efforts in modeling ORN have used limited size and single-institution data sets^7^ and lacked recommended external validation,^30^ potentially leading to overoptimistic single-institution models. The low prevalence of ORN represents a statistical challenge that limits trust and the successful adoption of existing research findings in the clinic. The existence of multiple coexisting ORN classification systems adds complexity to multi-institutional efforts.^31^ Although the PREDMORN consortium was developed to address these limitations, and this study represents a significant advancement in this direction, future work will pursue prospective validation of our results.

Finally, this study adopted a more traditional approach to modeling ORN to establish a baseline that enables comparison to existing publications. The PREDMORN study has been planned in 2 phases: although the first phase has used DVH data, the second phase will focus on higher dimensionality (imaging) data to incorporate spatial information. Future work will also explore other statistical options that do not assume linearity and address the inherent multicollinearity of DVH-based parameters.^32^

## Supplementary Material

Appendix. Supplementary materials

Supplementary material associated with this article can be found in the online version at doi:10.1016/j.ijrobp.2025.12.044.

## Figures and Tables

**Table 1:** Cohort characteristics for matched data sets (ie, aggregated data from institutions A to G) and whole population data set (ie, data from institution H only)

Cohort characteristics institutions A-G		
Variable	Control group (N = 848)	ORN group (N = 415)
Sex, N (%)		
Male/female	643 (75.8)/205 (24.2)	319 (76.9)/96 (23.1)
Age (y)		
NA, N (%)	0 (0.0)	1 (0.2)
Median (IQR)	60.1 (54.0–67.9)	61.0 (54.9–66.7)
Follow-up time (y)		
NA, N (%)	0 (0.0)	0 (0.0)
Median (IQR)	4.4 (2.7–5.7)	4.6 (3.1–6.0)
Primary site group, N (%)		
NA	0 (0.0)	0 (0.0)
OPC	546 (64.4)	249 (60.0)
OCC	231 (27.2)	129 (31.1)
Larynx/hypopharynx	44 (5.2)	19 (4.6)
Other	27 (3.2)	18 (4.3)
T stage, N (%)		
NA	0 (0.0)	2 (0.5)
TX/T0	5 (0.6)/13 (1.5)	1 (0.2)/6 (1.4)
T1	149 (17.6)	57 (13.7)
T1c	1 (0.1)	0 (0.0)
T2	278 (32.8)	136 (32.8)
T2b	1 (0.1)	0 (0.0)
T3	165 (19.5)	73 (17.6)
T4	105 (12.4)	90 (21.7)
T4a	119 (14.0)	43 (10.4)
T4b	11 (1.3)	7 (1.7)
N stage, N (%)		
NA	2 (0.2)	2 (0.5)
NX/N0	3 (0.4)/217 (25.6)	0 (0.0)/94 (22.7)
N1	119 (14.0)	49 (11.8)
N2	98 (11.6)	71 (17.1)
N2a	40 (4.7)	11 (2.7)
N2b	242 (28.5)	118 (28.4)
N2c	106 (12.5)	57 (13.7)
N3	21 (2.5)	13 (3.1)
M stage, N (%)		
NA	323 (38.1)	160 (38.6)
M0	522 (61.6)	255 (61.4)
M1	3 (0.4)	0 (0.0)
Mandible volume (cc)		
NA, N (%)	1 (0.1)	3 (0.7)
Median (IQR)	69.6 (56.8–83.1)	72.5 (60.6–87.6)
HPV, N (%)		
NA	735 (86.7)	372 (89.6)
HPV−	23 (2.7)	14 (3.4)
HPV+	90 (10.6)	29 (7.0)
Smoking status, N (%)		
NA	1 (0.1)	0 (0.0)
Current	259 (30.5)	179 (43.1)
Former	328 (38.7)	141 (34.0)
Never	260 (30.7)	95 (22.9)
Smoking amount, N (%)		
NA	298 (35.1)	139 (33.5)
>10 pack years	251 (29.6)	150 (36.1)
<10 pack years	90 (10.6)	41 (9.9)
None	209 (24.6)	85 (20.5)
Tobacco pack years		
NA, N (%)	480 (56.6)	230 (55.4)
Median (IQR)	3.0 (0.0–30.5)	10.0 (0.0–34.0)
Alcohol status, N (%)		
NA	398 (46.9)	205 (49.4)
Current	316 (37.3)	156 (37.6)
Former	47 (5.5)	15 (3.6)
Never	87 (10.3)	39 (9.4)
Pre-RT extractions, N (%)		
NA	10 (1.2)	0 (0.0)
Yes	362 (42.7)	207 (49.9)
No	476 (56.1)	208 (50.1)
Time extraction to RT (d)		
NA, N (%)	674 (79.5)	363 (87.5)
Median (IQR)	28.0 (20.0–47.0)	20.0 (14.0–31.5)
Chemotherapy, N (%)		
NA	1 (0.1)	4 (1.0)
Yes	544 (64.2)	285 (68.7)
No	303 (35.7)	126 (30.4)
RT technique, N (%)		
NA	0 (0.0)	0 (0.0)
IMRT	612 (72.2)	303 (73.0)
VMAT	236 (27.8)	112 (27.0)
Primary RT/PORT, N (%)		
NA	0 (0.0)	0 (0.0)
Primary RT	622 (73.3)	297 (71.6)
PORT	226 (26.7)	118 (28.4)
Fraction size, N (%)		
NA	0 (0.0)	0 (0.0)
>2 Gy/fraction	416 (49.1)	214 (51.6)
≤2 Gy/fraction	432 (50.9)	201 (48.4)
ECOG performance, N (%)		
NA	457 (53.9)	229 (55.2)
0	188 (22.2)	82 (19.8)
1	153 (18.0)	69 (16.6)
2	47 (5.5)	33 (8.0)
3	3 (0.4)	1 (0.2)
4	0 (0.0)	1 (0.2)
5	0 (0.0)	0 (0.0)
Xerostomia baseline, N (%)		
NA	650 (76.7)	325 (78.3)
0	166 (19.6)	81 (19.5)
1	30 (3.5)	8 (1.9)
2	0 (0.0)	1 (0.2)
3	0 (0.0)	0 (0.0)
4	4 (0.5)	0 (0.0)
Xerostomia 1-y post-RT, N (%)		
NA	543 (64.0)	265 (63.9)
0	26 (3.1)	17 (4.1)
1	92 (10.8)	59 (14.2)
2	182 (21.5)	71 (17.1)
3	5 (0.6)	3 (0.7)
4	0 (0.0)	0 (0.0)
*D*_mean_ parotids (Gy)		
NA, N (%)	652 (76.9)	337 (81.2)
Median (IQR)	26.3 (18.6–33.2)	27.8 (20.6–34.1)
Time to ORN (y)		
NA, N (%)	n/a	11 (2.7)
Median (IQR)		1.2 (0.6–2.3)
ORN grade Notani, N (%)		
NA	n/a	201 (48.4)
1		77 (18.6)
2		62 (14.9)
3		75 (18.1)
ORN grade CTCAE, N (%)		
NA	n/a	268 (64.6)
1		16 (3.9)
2		58 (14.0)
3		73 (17.6)
4		1 (0.2)
5		0 (0.0)
ORN grade Tsai, N (%)		
NA	n/a	215 (51.8)
1		24 (5.8)
2		51 (12.3)
3		60 (14.5)
4		65 (15.7)
Cohort characteristics institution H		
Variable	Control group (N = 2472)	ORN group (N = 215)
Sex, N (%)		
Male/female	653 (26.4)/1819 (73.6)	59 (27.4)/156 (72.6)
Age (y)		
NA, N (%)	0 (0.0)	0 (0.0)
Median (IQR)	61.3 (53.1–69.7)	59.0 (53.7–65.8)
Follow-up time (y)		
NA, N (%)	0 (0.0)	0 (0.0)
Median (IQR)	4.8 (1.9–6.3)	5.9 (4.1–8.2)
Primary site group, N (%)		
NA	0 (0.0)	0 (0.0)
Oropharynx	858 (34.7)	109 (50.7)
Oral cavity	414 (16.7)	73 (34.0)
Larynx/hypopharynx	286 (11.6)	3 (1.4)
Other	679 (27.5)	24 (11.2)
T stage, N (%)		
NA	29 (1.2)	0 (0.0)
TX	2 (0.1)	0 (0.0)
T0	211 (8.5)	13 (6.0)
T1	423 (17.1)	33 (15.3)
T1a	9 (0.4)	0 (0.0)
T1b	9 (0.4)	0 (0.0)
T1c	0 (0.0)	0 (0.0)
T2	664 (26.9)	71 (33.0)
T2b	9 (0.4)	0 (0.0)
T3	567 (22.9)	41 (19.1)
T3a	1 (0.0)	0 (0.0)
T4	83 (3.4)	2 (0.9)
T4a	367 (14.8)	50 (23.3)
T4b	93 (3.8)	5 (2.3)
Tis	5 (0.2)	0 (0.0)
N stage, N (%)		
NA	7 (0.3)	0 (0.0)
N0	694 (28.1)	60 (27.9)
NX	22 (0.9)	0 (0.0)
N1	319 (12.9)	26 (12.1)
N1a	1 (0.0)	0 (0.0)
N1b	7 (0.3)	0 (0.0)
N2	107 (4.3)	4 (1.9)
N2a	94 (3.8)	5 (2.3)
N2b	716 (29.0)	72 (33.5)
N2c	355 (14.4)	43 (20.0)
N3	121 (4.9)	5 (2.3)
N3a	9 (0.4)	0 (0.0)
N3b	20 (0.8)	0 (0.0)
M stage, N (%)		
NA	29 (1.2)	0 (0.0)
M0	2440 (98.7)	215 (100.0)
M1	3 (0.1)	0 (0.0)
Mandible volume (cc)		
NA, N (%)	NA	NA
Median (IQR)		
HPV, N (%)		
NA	1296 (52.4)	94 (43.7)
HPV−	403 (34.0)	40 (33.0)
HPV+	773 (66.0)	81 (67.0)
Smoking status, N (%)		
NA	71 (2.9)	2 (0.9)
Current	709 (28.7)	87 (40.5)
Former	791 (32.0)	70 (32.6)
Never	901 (36.4)	56 (26.0)
Smoking amount, N (%)		
NA	70 (2.8)	3 (1.4)
>10 pack (y)	1181 (47.8)	73 (34.0)
<10 pack (y)	1221 (49.4)	139 (64.7)
None	901 (36.4)	56 (26.0)
Alcohol status, N (%)		
NA	153 (6.2)	8 (3.7)
Current	1187 (48.0)	127 (59.1)
Former	177 (7.2)	17 (7.9)
Never	955 (38.6)	63 (29.3)
Pre-RT extractions, N (%)		
NA	1 (0.0)	0 (0.0)
Yes	860 (34.8)	83 (38.6)
No	1611 (65.2)	132 (61.4)
Time extraction to RT (d)		
NA, N (%)	NA	NA
Median (IQR)		
Chemotherapy, N (%)		
NA	0 (0.0)	0 (0.0)
Yes	1057 (42.8)	103 (47.9)
No	1415 (57.2)	112 (52.1)
RT technique, N (%)		
NA	NA	NA
IMRT		
VMAT		
Primary RT/PORT, N (%)		
NA	0 (0.0)	0 (0.0)
Primary RT	1582 (64.0)	139 (64.7)
PORT	890 (36.0)	76 (35.3)
Fraction size, N (%)		
NA	NA	NA
>2 Gy/fraction		
≤2 Gy/fraction		
ECOG PS, N (%)		
NA	26 (1.1)	0 (0.0)
0	1402 (56.7)	131 (60.9)
1	901 (36.4)	78 (36.3)
2	119 (4.8)	6 (2.8)
3	24 (1.0)	0 (0.0)
4	0 (0.0)	0 (0.0)
5	0 (0.0)	0 (0.0)
Xerostomia baseline, N (%)		
NA	NA	NA
0		
1		
2		
3		
4		
Xerostomia 1-y post-RT, N (%)		
NA	NA	NA
0		
1		
2		
3		
4		
D_mean_ parotids (Gy)		
NA, N (%)	NA	NA
Median (IQR)		
Time to ORN (y)		
NA, N (%)	n/a	4 (1.9)
Median (IQR)		1.4 (0.6, 3.1)
ORN grade Notani, N (%)		
NA	n/a	48 (22.3)
1		113 (52.6)
2		40 (18.6)
3		14 (6.5)
ORN grade CTCAE, N (%)		
NA	n/a	49 (22.8)
1		37 (17.2)
2		121 (56.3)
3		8 (3.7)
4		0 (0.0)
5		0 (0.0)
ORN grade Tsai, N (%)		
NA	n/a	85 (39.5)
1		67 (31.2)
2		49 (22.8)
3		6 (2.8)
4		8 (3.7)

*Abbreviations:* CTCAE = common terminology criteria for adverse events; ECOG PS = eastern cooperative oncology group performance status; HPV = human papillomavirus; IMRT = intensity modulated radiation therapy; IQR = interquartile range; OCC = oral cavity cancer; OPC = oropharyngeal cancer; ORN = osteoradionecrosis; PORT = postoperative RT; RT = radiation therapy; VMAT = volumetric modulated arc therapy.

See tables B1 and B2 for subset and institution-specific descriptive tables. NA indicates missing data (not available); n/a indicates “not applicable.”

**Table 2:** ORN NTCP model parameters

Variables	β	OR (95% CI)	*P*value
Intercept	−3.749		
D_30%_ (Gy)	0.048*	1.05* (1.03–1.07)	<.001
Pre-RT dental extraction (yes vs no)	0.481	1.62 (1.21–2.15)	.001
V_70Gy_ (%)	0.025*	1.03* (1.00–1.05)	.024
Smoking status (current vs never/former)	0.333	1.39 (1.03–1.87)	.026

*Abbreviations:* OR = odds ratio; ORN NTCP = osteoradionecrosis normal tissue complication probability; RT = radiation therapy.

* The *β* and OR values are expressed per unit increase in these variables.

**Table 3:** Model performance results of the ORN NTCP model on the training, test, and the 2 external validation data sets

Metric	Training (N = 948)	Test (N = 236)	External validation 1 (N = 58)	External validation 2 (N = 2687)
ROC AUC (95% CI)	0.67 (0.64–0.71)	0.69 (0.62–0.76)	0.69 (0.53–0.84)	0.65 (0.61–0.69)
Nagelkerke *R*^2^	0.108	0.122	0.098	0.046
Brier score	0.203	0.204	0.199	0.077
Log Loss	0.591	0.594	0.596	0.281

*Abbreviations:* AUC, area under the curve; ORN NTCP, osteoradionecrosis normal tissue complication probability; ROC, receiver operating characteristic.

The 95% CI for the area under the ROC curve (AUC) has been calculated using the DeLong method.

## Data Availability

Research data are stored in an institutional repository and will be shared upon reasonable request to the corresponding author and subject to a Material Transfer Agreement (MTA) with each participating institution individually.
