# Supplementary material for: Multi-institutional Normal Tissue Complication Probability (NTCP) Prediction Model for Mandibular Osteoradionecrosis: Results from the PREDMORN Study
Source: Int J Radiat Oncol Biol Phys. Author manuscript; Available in PMC 2026 Feb 25. (PMC12930408; doi:10.1016/j.ijrobp.2025.12.044)
Supplement: Appendix. Supplementary materials [file NIHMS2148559-supplement-Appendix__Supplementary_materials.pdf]

Supplementary Material - Table of Contents

Supplement A. Study design

Supplement B. Clinical and demographic data

Supplement C. Univariable analyses

Supplement D. Mandible volume analyses

Supplement E. Dosimetric data

Supplement F. Time to ORN analyses

Supplement G. Multivariable stepwise forward logistic regression analyses

Supplement H. Sub-cohort analyses

Supplement I. EQD2 approximation

Supplement J. TRIPOD checklist

Supplement A. Study design

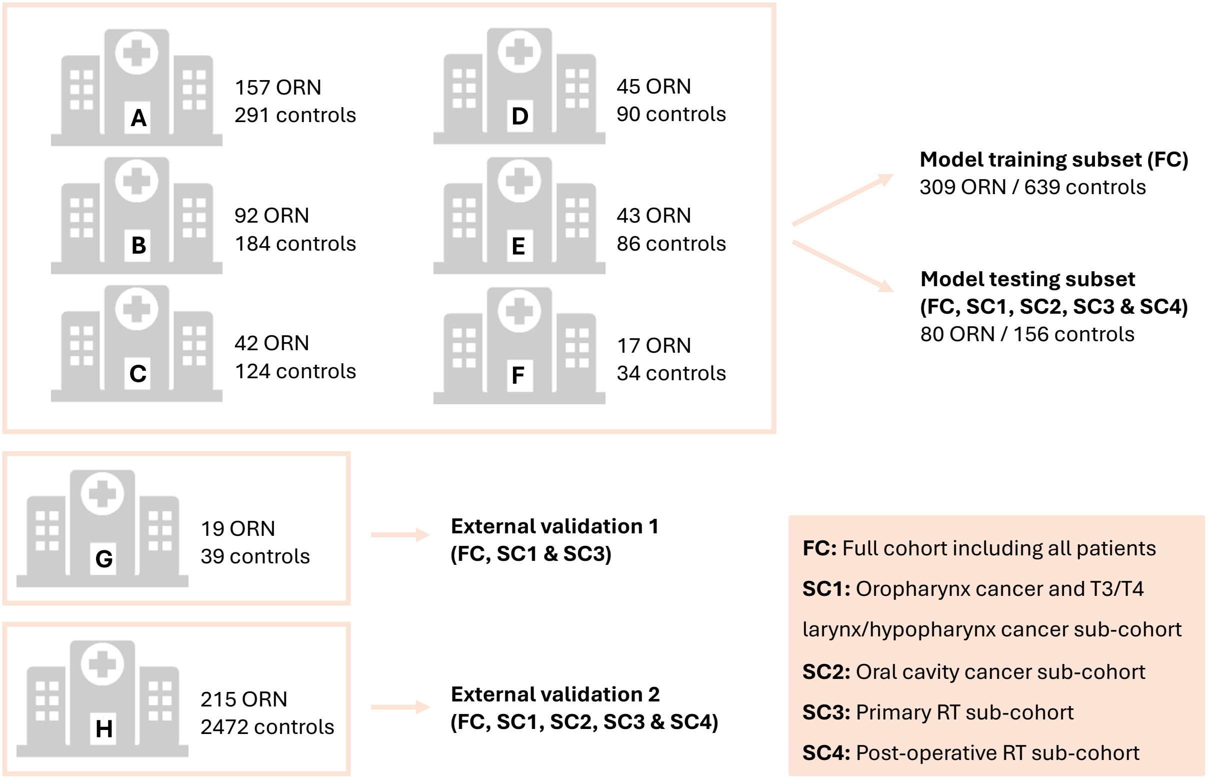

Figure A1. Schematics of the study design. Data from six institutions (A to F) was used to train (80%) and test (20%) the NTCP model. This model was then externally validated twice using two separate independent datasets (G and H). The NTCP model was tested and externally validated on a full cohort (FC) that included all patients as well as on sub-cohorts (SC) of specific primary tumour sites (SC1 and SC2) or treatment approaches (SC3 and SC4).

Table A1: Inclusion and exclusion criteria for the study as defined in the protocol [1].

**Inclusion criteria**	• HNC cases treated radically with RT or chemo-radiation (CRT) and post-operative RT (PORT)+/- chemotherapy (C-PORT) using IMRT or VMAT.<br>*Specific to ORN cases:*<br>• Confirmed diagnosis of ORN (any grade). ORN defined clinically as 'an area of exposed bone in the mandible that had been present for at least 8 weeks in a previously irradiated field, in the absence of recurrent tumour' [20]. NB: cases with recurrences outside of the HN region will be included.<br>*Specific to control cases:*<br>• Any histology except for T1/2N0 Larynx cases, as these do not receive significant dose to the mandible.<br>• The selection of controls should be as per the methodology described in Figure 1.
**Exclusion criteria**	• Datasets without available CT volume, RT structure or RT dose DICOM files.<br>• ORN outside the mandible (e.g. maxilla). NB: if multiple ORN sites, cases will be included if at least one of the sites is the mandible.<br>• Cases with re-irradiation to the HN region.<br>• Cases treated with less than a radical dose of radiation and/or those with life expectancy at diagnosis less than 12 months.

Table A2: Variables for data collection as proposed in the protocol [1].

Variable	Units/format/options	Comments
Sex	male/female	
Date/year[1] of birth	dd/mm/yyyy	This is needed to calculate the age at the start of RT
RT start date/year	dd/mm/yyyy	
RT end date	dd/mm/yyyy	
Date/year of last follow-up	dd/mm/yyyy	This is needed to calculate the follow-up time since the end of RT
Primary site group	• oral cavity (OCC)<br>• oropharynx (OPC)<br>• paranasal sinus/nasopharynx (PNS/NP)<br>• larynx/hypopharynx<br>• salivary gland<br>• unknown primary	
TNM stage	• T: X, 0, 1, 2, 3, 4, 4a, 4b<br>• N: 0, 1, 2, 2a, 2b, 2c, 3<br>• M: 0, 1, M	Use of TNM7 notation
HPV	• HPV+ / HPV- / not available	If available
Smoking status at start of RT	• Current: smoking at the start or within 3 months of the start of RT<br>• Previous: stopped smoking at least more than 3 months prior to the start of RT<br>• Never: the patient has never smoked	If available
Smoking amount at start of RT	• None / < 10 pack years / > 10 pack years / not available	If available
Alcohol status at start of RT	Current: alcohol intake at the start or within 3 months of the start of RT. If available, also state amount: < 21 u/w or > 21 u/w. / Previous: stopped alcohol intake at least more than 3 months prior to the start of RT. / Never: no alcohol intake at all	If available
ECOG performance status at start of RT	• 0: Able to carry normal activity without restriction<br>• 1: Restricted in physically strenous activity but ambulatory and able to carry out light work<br>• 2: Ambulatory and capable of self-care but unable to carry out any work. Up and about for more than 50% of waking hours<br>• 3: Capable only of limited self care; confined to bed or chair more than 50% of waking hours<br>• 4: Completely disabled; cannot carry out any self care; totally confined to bed or chair<br>• Not available	If available
Xerostomia at baseline	• G0: None<br>• G1 (Mild): Symptomatic (e.g., dry or thick saliva) without significant dietary alteration; unstimulated saliva flow >0.2ml/min<br>• G2 (Moderate): Oral intake alterations (e.g., copious water, other lubricants, diet limited to purees or soft food); unstimulated saliva flow 0.1-0.2 ml/min<br>• G3 (Severe): Inability to adequately aliment orally; tube feedings or TPN indicated, unstimulated saliva flow <0.1 ml/min<br>• not available	If available. Use the NCI CTCAE v4/v5 grading system (both versions are the same)
Xerostomia (grade≥2 at ≥1year post-RT)	Use NCI CTCAE v4/v5 grading system described above or the RTOG grading system for late toxicities described for ORN below.	If available. NCI CTCAE v4/v5 or RTOG
Pre-RT dental assessment	Yes / No	

Pre-RT dental extraction	Yes / No	
Teeth extracted	If any extractions performed, number the teeth extracted according to the FDI system	Use the FDI (World Dental Federation) numbering system
Date of pre-RT dental extraction	dd/mm/yyyy	This is needed to calculate the time from extraction to RT start
Pre-RT surgery	Primary RT (no pre-RT surgery) / Post-operative RT (PORT)	
Pre-RT surgery type	If available, describe the type and site of surgery	
RT technique	IMRT / VMAT	Cases treated with 3D conformal RT are excluded from the study
TPS	Specify radiotherapy treatment planning system and algorithm used to produce the clinical treatment plan (e.g. Monaco, Monte Carlo, Eclipse-Acuros XB, Eclipse-AAA, etc.)	This is needed in order to anticipate variations in exported data details between TPS
Dose calculation and reporting	Dm,m / Dw,m / Dw,w	This is needed to identify potential systematic biases in reported absorbed dose
Prescribed dose	in Gy	
Total number of fractions		This is needed to calculate the fraction size
Treatment schedule	Please specify number of fractions per week and how these are distributed (e.g. 5 fractions/week, 1 fraction/day)	
Delivered dose and fractions		If different from prescribed
Mandible volume	in cc	
Dmean parotid glands	in Gy	
Dmean submandibular glands	in Gy	
Chemotherapy type	None / Carboplatin / Cisplatin / Cetuximab / Other	
ORN	Yes / No	The info below is only relevant to ORN cases
Date or ORN diagnosis	dd/mm/yyyy	This is needed to calculate the time to ORN diagnosis since the end of RT
ORN site	Specify using the notation based upon equivalent teeth	
Primary ORN cause	Spontaneous / Induced - pre-RT dental extraction / Induced - post-RT dental extractions / Induced - dental infection / Induced - dental implant / Induced – denture / Induced - mandible plate infection	
ORN grade (Notani) at diagnosis	Stage I: ORN confined to alveolar bone / Stage II: ORN limited to the alveolar bone and/or above the level of the inferior alveolar canal / Stage III: ORN under the lower part of the inferior alveolar canal, with fistula or bone fracture	Use the Notani grading system
Peak ORN grade (Notani)	(same as above)	Use the Notani grading system
Date of peak grade (Notani)	dd/mm/yyyy	
ORN grade (CTCAE v4) at diagnosis	• Grade 1: Asymptomatic; clinical or diagnostic observations only; intervention not indicated<br>• Grade 2: Symptomatic; medical intervention indicated (e.g., topical agents); limiting instrumental ADL<br>• Grade 3: Severe symptoms; limiting self-care ADL; elective operative intervention indicated<br>• Grade 4: Life-threatening consequences; urgent intervention indicated<br>• Grade 5: Death	Use the CTCAE v4 grading system
Peak ORN grade (CTCAE v4)	(same as above)	Use the CTCAE v4 grading system
Date of peak grade (CTCAE v4)	dd/mm/yyyy	

Supplement B. Clinical and demographic data

Table B1: Clinical and demographic characteristics of the train, test and external validation datasets. NA indicates missing data ('not available'); n/a indicates 'not applicable'. Variables with a * symbol next to them were selected for statistical analyses. Variation across train/test/external datasets for these variables was assessed using ANOVA or chi-squared tests, as appropriate.

Variables		p	Train dataset		Test dataset	
			**Control (N=639)**	**ORN (N=309)**	**Control (N=156)**	**ORN (N=80)**
Sex* (N, %)	*NA*	<0.001	0 (0.0)	0 (0.0)	0 (0.0)	0 (0.0)
	Male		473 (74.0)	236 (76.4)	125 (80.1)	65 (81.3)
	Female		166 (26.0)	73 (23.6)	31 (19.9)	15 (18.8)
Age* (years)	*NA (N, %)*	0.657	0 (0.0)	0 (0.0)	0 (0.0)	0 (0.0)
	median (IQR)		61.0 (54.0-68.0)	61 (55.0-66.9)	60 (54.2-66.9)	61 (54.0-67.0)
Follow-up time (years)	*NA (N, %)*		0 (0.0)	0 (0.0)	0 (0.0)	0 (0.0)
	median (IQR)		4.4 (2.7-5.8)	4.7 (3.2-6.1)	4.1 (3.0-5.5)	5.0 (3.2-6.0)
Primary Site Group (N, %)	*NA*		5 (0.8)	7 (2.3)	4 (2.6)	1 (1.3)
	Oropharynx		414 (64.8)	185 (59.9)	102 (65.4)	53 (66.3)
	Oral cavity		182 (28.5)	96 (31.1)	41 (26.3)	22 (27.5)
	Larynx/hypopharynx		21 (3.3)	11 (3.6)	4 (2.6)	2 (2.5)
	Other		17 (2.7)	10 (3.2)	5 (3.2)	2 (2.5)
T stage (N, %)	*NA*		6 (0.9)	4 (1.3)	3 (1.9)	2 (2.5)
	TX		0 (0.0)	0 (0.0)	0 (0.0)	0 (0.0)
	T0		6 (0.9)	1 (0.3)	2 (1.3)	0 (0.0)
	T1		115 (18.0)	44 (14.2)	30 (19.2)	8 (10.0)
	T1c		0 (0.0)	0 (0.0)	0 (0.0)	0 (0.0)
	T2		201 (31.5)	104 (33.7)	53 (34.0)	29 (36.3)
	T2b		0 (0.0)	0 (0.0)	0 (0.0)	0 (0.0)
	T3		121 (18.9)	52 (16.8)	29 (18.6)	14 (17.5)
	T4		190 (29.7)	104 (33.7)	39 (25.0)	27 (33.8)
	T4a		0 (0.0)	0 (0.0)	0 (0.0)	0 (0.0)
	T4b		0 (0.0)	0 (0.0)	0 (0.0)	0 (0.0)
N stage (N, %)	*NA*		1 (0.2)	1 (0.3)	1 (0.6)	1 (1.3)
	N0		170 (26.6)	64 (20.7)	37 (23.7)	21 (26.3)
	NX		0 (0.0)	0 (0.0)	0 (0.0)	0 (0.0)
	N1		80 (12.5)	34 (11.0)	30 (19.2)	14 (17.5)
	N2		373 (58.4)	199 (64.4)	83 (53.2)	44 (55.0)
	N2a		0 (0.0)	0 (0.0)	0 (0.0)	0 (0.0)
	N2b		0 (0.0)	0 (0.0)	0 (0.0)	0 (0.0)
	N2c		0 (0.0)	0 (0.0)	0 (0.0)	0 (0.0)
	N3		15 (2.3)	11 (3.6)	5 (3.2)	0 (0.0)
M stage (N, %)	*NA*		253 (39.6)	124 (40.1)	68 (43.6)	32 (40.0)
	M0		383 (60.4)	185 (59.9)	88 (56.4)	48 (60.0)
	M1		3 (0.5)	0 (0.0)	0 (0.0)	0 (0.0)
Mandible volume (cc)*	*NA (N, %)*	0.011	0 (0.0)	0 (0.0)	0 (0.0)	0 (0.0)
	median (IQR)		70.4 (56.6-84.3)	72.5 (60.6-87.7)	69.7 (59.0-83.5)	74.9 (63.4-90.9)
HPV (N, %)	*NA*		537 (84.0)	267 (86.4)	126 (80.8)	65 (81.3)
	HPV-		42 (6.6)	21 (6.8)	26 (16.7)	9 (11.3)
	HPV+		60 (9.4)	21 (6.8)	4 (2.6)	6 (7.5)
Smoking status (N, %)*	*NA*	<0.001	0 (0.0)	0 (0.0)	0 (0.0)	0 (0.0)
	Current		203 (31.8)	128 (41.4)	46 (29.5)	37 (46.3)
	Former		234 (36.6)	107 (34.6)	71 (45.5)	28 (35.0)
	Never		202 (31.6)	74 (23.9)	39 (25.0)	15 (18.8)
Smoking amount (N, %)	*NA*		235 (36.8)	100 (32.4)	49 (31.4)	27 (33.8)
	> 10 pack years		173 (27.1)	105 (34.0)	56 (35.9)	29 (36.3)
	< 10 pack years		59 (9.2)	30 (9.7)	12 (7.7)	9 (11.3)
	None		172 (26.9)	74 (23.9)	39 (25.0)	15 (18.8)
Alcohol status (N, %)	*NA*		380 (59.5)	193 (62.5)	87 (55.8)	47 (58.8)
	Current		179 (28.0)	80 (25.9)	46 (29.5)	29 (36.3)
	Former		27 (4.2)	12 (3.9)	12 (7.7)	2 (2.5)
	Never		53 (8.3)	24 (7.8)	11 (7.1)	2 (2.5)
Pre-RT extractions (N, %)*	*NA*	0.031	0 (0.0)	0 (0.0)	0 (0.0)	0 (0.0)
	Yes		270 (42.3)	162 (52.4)	75 (48.1)	41 (51.3)
	No		369 (57.7)	147 (47.6)	81 (51.9)	39 (48.8)
Time extraction to RT (days)	*NA (N, %)*		369 (100)	269 (87.1)	123 (78.8)	70 (87.5)
	median (IQR)		30.0 (20.5-49.0)	20.0 (16.3-33.0)	28.0 (24.0-43.0)	26.5 (18.5-29.0)

Chemotherapy	*NA*		0 (0.0)	0 (0.0)	0 (0.0)	0 (0.0)
(N, %)*	*Yes*	<0.001	402 (62.9)	215 (69.6)	98 (62.8)	53 (66.3)
	No		237 (37.1)	94 (30.4)	58 (37.2)	27 (33.8)
RT technique	*NA*		0 (0.0)	0 (0.0)	0 (0.0)	0 (0.0)
(N, %)*	*IMRT*	<0.001	478 (74.8)	235 (76.1)	122 (78.2)	61 (76.3)
	VMAT		161 (25.2)	74 (23.9)	34 (21.8))	19 (23.8)
Primary	*NA*		0 (0.0)	0 (0.0)	0 (0.0)	0 (0.0)
RT/PORT	*Primary RT*	<0.001	460 (72.0)	219 (70.9)	117 (75.0)	59 (73.8)
(N, %)*	*PORT*		179 (28.0)	90 (29.1)	39 (25.0)	21 (26.3)
Fraction size	*NA*		0 (0.0)	0 (0.0)	0 (0.0)	0 (0.0)
(N, %)*	*> 2Gy/fraction*	<0.001	321 (50.2)	164 (53.1)	84 (53.8)	47 (58.8)
	<= 2Gy/fraction		318 (49.8)	145 (46.9)	72 (46.2)	33 (41.3)
ECOG PS	*NA*		341 (53.4)	166 (53.7)	73 (46.8)	46 (57.5)
(N, %)	*0*		139 (21.8)	65 (21.0)	42 (26.9)	12 (15.0)
	1		119 (18.6)	55 (17.8)	31 (19.9)	11 (13.8)
	2		38 (5.9)	22 (7.1)	9 (5.8)	11 (13.8)
	3		2 (0.3)	1 (0.3)	1 (0.6)	0 (0.0)
	4		0 (0.0)	0 (0.0)	0 (0.0)	0 (0.0)
	5		0 (0.0)	0 (0.0)	0 (0.0)	0 (0.0)
Xerostomia	*NA*		526 (82.3)	251 (81.2)	122 (78.2)	65 (81.3)
baseline	*0*		99 (15.5)	54 (17.5)	30 (19.2)	14 (17.5)
(N, %)	*1*		14 (2.2)	4 (1.3)	4 (2.6)	1 (1.3)
	2		0 (0.0)	0 (0.0)	0 (0.0)	0 (0.0)
	3		0 (0.0)	0 (0.0)	0 (0.0)	0 (0.0)
	4		0 (0.0)	0 (0.0)	0 (0.0)	0 (0.0)
Xerostomia 1-	*NA*		414 (64.8)	196 (63.4)	99 (63.5)	52 (65.0)
year post-RT	*0*		17 (2.7)	10 (3.2)	5 (3.2)	5 (6.3)
(N, %)	*1*		208 (32.6)	103 (33.3)	52 (33.3)	23 (28.8)
	2		0 (0.0)	0 (0.0)	0 (0.0)	0 (0.0)
	3		0 (0.0)	0 (0.0)	0 (0.0)	0 (0.0)
	4		0 (0.0)	0 (0.0)	0 (0.0)	0 (0.0)
Dmean	*NA (N, %)*		512 (80.1)	266 (86.1)	127 (81.4)	64 (80.0)
parotids	*median (IQR)*		24.5	24.2	26.1	22.0
(Gy)			(16.8-32.9)	(21.1-33.6)	(18.6-32.5)	(15.7-30.4)
Time to ORN	*NA (N, %)*			8 (2.6)		3 (3.8)
(years)	*median (IQR)*		n/a	1.2	n/a	1.1
				(0.6-2.4)		(0.5-1.8)
ORN grade	*NA*			156 (50.5)		41 (51.3)
Notani	*1*		n/a	61 (19.7)	n/a	11 (13.8)
(N, %)	*2*			38 (12.3)		11 (13.8)
	3			54 (17.5)		17 (21.3)
ORN grade	*NA*			195 (63.1)		48 (60.0)
CTCAE	*1*			12 (3.9)		3 (3.8)
(N, %)	*2*		n/a	48 (15.5)	n/a	10 (12.5)
	3			54 (17.5)		18 (22.5)
	4			0 (0.0)		1 (1.3)
	5			0 (0.0)		0 (0.0)
ORN grade	*NA*			187 (60.5)		48 (60.0)
Tsai	*1*			17 (5.5)		4 (5.0)
(N, %)	*2*		n/a	22 (7.1)	n/a	11 (13.8)
	3			39 (12.6)		8 (10.0)
	4			44 (14.2)		9 (11.3)

Table B1 (continued)

**Variables**		**External dataset 1 (Institution G)**		**External dataset 2 (Institution H)**	
		**Control (N=39)**	**ORN (N=19)**	**Control (N=2472)**	**ORN (N=215)**
Sex*	*NA*	0 (0.0)	0 (0.0)	0 (0.0)	0 (0.0)
(N, %)	*Male*	34 (87.2)	14 (73.7)	653 (26.4)	59 (27.4)
	Female	5 (12.8)	5 (26.3)	1819 (73.6)	156 (72.6)
Age*	*NA (N, %)*	0 (0.0)	0 (0.0)	0 (0.0)	0 (0.0)
(years)	*median (IQR)*	61.5 (56.3-70.0)	58.9 (55.5-64.2)	61.3 (53.1, 69.7)	59.0 (53.7, 65.8)
Follow-up	*NA (N, %)*	0 (0.0)	0 (0.0)	0 (0.0)	0 (0.0)
time	*median (IQR)*	4.8 (1.7-6.1)	3.7 (2.4-4.7)	4.8 (1.9, 6.3)	5.9 (4.1, 8.2)
(years)					
Primary Site	*NA*	0 (0.0)	0 (0.0)	0 (0.0)	0 (0.0)
Group	*Oropharynx*	22 (56.4)	10 (52.6)	858 (34.7)	109 (50.7)
(N, %)	*Oral cavity*	2 (5.1)	5 (26.3)	414 (16.7)	73 (34.0)
	Larynx/hypopharynx	10 (25.6)	1 (5.3)	286 (11.6)	3 (1.4)
	Other	5 (12.8)	3 (15.8)	679 (27.5)	24 (11.2)
T stage	*NA*	0 (0.0)	0 (0.0)	29 (1.2)	0 (0.0)

(N, %)	*TX*	1 (2.6)	1 (5.3)	2 (0.1)	0 (0.0)
	T0	0 (0.0)	1 (5.3)	211 (8.5)	13 (6.0)
	T1	3 (7.7)	5 (26.3)	423 (17.1)	33 (15.3)
	T1a	0 (0.0)	0 (0.0)	9 (0.4)	0 (0.0)
	T1b	0 (0.0)	0 (0.0)	9 (0.4)	0 (0.0)
	T1c	0 (0.0)	0 (0.0)	0 (0.0)	0 (0.0)
	T2	17 (43.6)	3 (15.8)	664 (26.9)	71 (33.0)
	T2b	0 (0.0)	0 (0.0)	9 (0.4)	0 (0.0)
	T3	15 (38.5)	5 (26.3)	567 (22.9)	41 (19.1)
	T3a	0 (0.0)	0 (0.0)	1 (0.0)	0 (0.0)
	T4	3 (7.7)	4 (21.1)	83 (3.4)	2 (0.9)
	T4a	0 (0.0)	0 (0.0)	367 (14.8)	50 (23.3)
	T4b	0 (0.0)	0 (0.0)	93 (3.8)	5 (2.3)
	Tis	0 (0.0)	0 (0.0)	5 (0.2)	0 (0.0)
N stage	*NA*	0 (0.0)	0 (0.0)	7 (0.3)	0 (0.0)
(N, %)	*N0*	8 (20.5)	6 (31.6)	694 (28.1)	60 (27.9)
	NX	0 (0.0)	0 (0.0)	22 (0.9)	0 (0.0)
	N1	8 (20.5)	1 (5.3)	319 (12.9)	26 (12.1)
	N1a	0 (0.0)	0 (0.0)	1 (0.0)	0 (0.0)
	N1b	0 (0.0)	0 (0.0)	7 (0.3)	0 (0.0)
	N2	9 (23.1)	6 (31.6)	107 (4.3)	4 (1.9)
	N2a	2 (5.1)	1 (5.3)	94 (3.8)	5 (2.3)
	N2b	5 (12.8)	3 (15.8)	716 (29.0)	72 (33.5)
	N2c	5 (12.8)	1 (5.3)	355 (14.4)	43 (20.0)
	N3	2 (5.1)	1 (5.3)	121 (4.9)	5 (2.3)
	N3a	0 (0.0)	0 (0.0)	9 (0.4)	0 (0.0)
	N3b	0 (0.0)	0 (0.0)	20 (0.8)	0 (0.0)
M stage	*NA*	0 (0.0)	0 (0.0)	29 (1.2)	0 (0.0)
(N, %)	*M0*	39 (100.0)	19 (100.0)	2440 (98.7)	215 (100.0)
	M1	0 (0.0)	0 (0.0)	3 (0.1)	0 (0.0)
Mandible	*NA (N, %)*	0 (0.0)	0 (0.0)		
volume (cc)*	*median (IQR)*	65.3	64.1 (	NA	NA
		(56.0-72.6)	60.6-70.8)		
HPV (N, %)	*NA*	15 (38.5)	7 (36.8)	1296 (52.4)	94 (43.7)
	HPV-	3 (7.7)	4 (21.1)	403 (34.0)	40 (33.0)
	HPV+	21 (53.8)	8 (42.1)	773 (66.0)	81 (67.0)
Smoking	*NA*	0 (0.0)	0 (0.0)	71 (2.9)	2 (0.9)
status	*Current*	7 (17.9)	10 (52.6)	709 (28.7)	87 (40.5)
(N, %)*	*Former*	18 (46.2)	5 (26.3)	791 (32.0)	70 (32.6)
	Never	14 (35.9)	4 (21.1)	901 (36.4)	56 (26.0)
Smoking	*NA*	4 (10.3)	1 (5.3%)	70 (2.8)	3 (1.4)
amount	*> 10 pack years*	19 (48.7)	14 (73.7)	1181 (47.8)	73 (34.0)
(N, %)	*< 10 pack years*	2 (5.1)	0 (0.0)	1221 (49.4)	139 (64.7)
	None	14 (35.9)	4 (21.1)	901 (36.4)	56 (26.0)
Alcohol	*NA*	14 (35.9)	0 (0.0)	153 (6.2)	8 (3.7)
status (N, %)	*Current*	16 (41.0)	11 (57.9)	1187 (48.0)	127 (59.1)
	Former	0 (0.0)	0 (0.0)	177 (7.2)	17 (7.9)
	Never	9 (23.1)	8 (42.1)	955 (38.6)	63 (29.3)
Pre-RT	*NA*	0 (0.0)	0 (0.0)	1 (0.0)	0 (0.0)
extractions	*Yes*	15 (38.5)	2 (10.5)	860 (34.8)	83 (38.6)
(N, %)*	*No*	24 (61.5)	17 (89.5)	1611 (65.2)	132 (61.4)
Time	*NA (N, %)*	26 (66.7)	17 (89.5)		
extraction to	*median (IQR)*	18.0	12.5	NA	NA
RT (days)		(15.0-32.0)	(8.3-16.8)		
Chemothera	*NA*	0 (0.0)	0 (0.0)	0 (0.0)	0 (0.0)
py (N, %)*	*Yes*	36 (92.3)	15 (78.9)	1057 (42.8)	103 (47.9)
	No	3 (7.7)	4 (21.1)	1415 (57.2)	112 (52.1)
RT	*NA*	0 (0.0)	0 (0.0)		
technique	*IMRT*	3 (7.7)	0 (0.0)	NA	NA
(N, %)*	*VMAT*	36 (92.3)	19 (100.0)		
Primary	*NA*	0 (0.0)	0 (0.0)	0 (0.0)	0 (0.0)
RT/PORT	*Primary RT*	36 (92.3)	18 (94.7)	1582 (64.0)	139 (64.7)
(N, %)*	*PORT*	3 (7.7)	1 (5.3)	890 (36.0)	76 (35.3)
Fraction size	*NA*	0 (0.0)	0 (0.0)		
(N, %)*	*> 2Gy/fraction*	2 (5.1)	2 (10.5)	NA	NA
	<= 2Gy/fraction	37 (94.9)	17 (89.5)		
ECOG PS	*NA*	39 (100.0)	13 (68.4)	26 (1.1)	0 (0.0)
(N, %)	*0*	NA	3 (15.8)	1402 (56.7)	131 (60.9)
	1		2 (10.5)	901 (36.4)	78 (36.3)
	2		0 (0.0)	119 (4.8)	6 (2.8)
	3		0 (0.0)	24 (1.0)	0 (0.0)
	4		1 (5.3)	0 (0.0)	0 (0.0)
	5		0 (0.0)	0 (0.0)	0 (0.0)
Xerostomia	*NA*	1 (2.6)	5 (26.3)	NA	
baseline	*0*	29 (74.4)	11 (57.9)		NA

(N, %)	*1*<br>*2*<br>*3*<br>*4*	9 (23.1)<br>0 (0.0)<br>0 (0.0)<br>0 (0.0)	3 (15.8)<br>0 (0.0)<br>0 (0.0)<br>0 (0.0)		
Xerostomia 1-year post-RT (N, %)	*NA*<br>*0*<br>*1*<br>*2*<br>*3*<br>*4*	25 (64.1)<br>4 (10.3)<br>10 (25.6)<br>0 (0.0)<br>0 (0.0)<br>0 (0.0)	11 (57.9)<br>2 (10.5)<br>6 (31.6)<br>0 (0.0)<br>0 (0.0)<br>0 (0.0)	NA	NA
Dmean parotids (Gy)	*NA (N, %)*<br>*median (IQR)*	0 (0.0)<br>29.1 (26.1-33.9)	0 (0.0)<br>33.3 (28.0-40.0)	NA	NA
Time to ORN (years)	*NA (N, %)*<br>*median (IQR)*	n/a	0 (0.0)<br>0.9 (0.6-1.7)	NA	4 (1.9)<br>1.4 (0.6, 3.1)
ORN grade Notani (N, %)	*NA*<br>*1*<br>*2*<br>*3*	n/a	0 (0.0)<br>4 (21.1)<br>12 (63.2)<br>3 (15.8)	n/a	48 (22.3)<br>113 (52.6)<br>40 (18.6)<br>14 (6.5)
ORN grade CTCAE (N, %)	*NA*<br>*1*<br>*2*<br>*3*<br>*4*<br>*5*	n/a	19 (100.0)	n/a	49 (22.8)<br>37 (17.2)<br>121 (56.3)<br>8 (3.7)<br>0 (0.0)<br>0 (0.0)
ORN grade Tsai (N, %)	*NA*<br>*1*<br>*2*<br>*3*<br>*4*	n/a	19 (100.0)	n/a	85 (39.5)<br>67 (31.2)<br>49 (22.8)<br>6 (2.8)<br>8 (3.7)

Table B2: Clinical and demographic characteristics by participating institution (institutions G and H are described above in Table B1). NA indicates missing data ('not available'); n/a indicates 'not applicable'. Variables with a * symbol next to them were selected for statistical analyses. Variation across centres for these variables was assessed using ANOVA or chi-squared tests, as appropriate.

Variables	p	Institution A		Institution B		Institution C		Institution D		Institution E		Institution F	
		**Control (N=291)**	**ORN (N=157)**	**Control (N=184)**	**ORN (N=92)**	**Control (N=124)**	**ORN (N=42)**	**Control (N=90)**	**ORN (N=45)**	**Control (N=86)**	**ORN (N=43)**	**Control (N=34)**	**ORN (N=17)**
Sex (N, %)*													
Male	<0.001	243 (83.5)	136 (86.6)	144 (78.3)	66 (71.7)	85 (68.5)	25 (59.5)	60 (66.7)	33 (73.3)	54 (62.8)	29 (67.4)	23 (67.6)	16 (94.1)
Female		48 (16.5)	21 (13.4)	40 (21.7)	26 (28.3)	39 (31.5)	17 (40.5)	30 (33.3)	12 (26.7)	32 (37.2)	14 (32.6)	11 (32.4)	1 (5.9)
Age (years)*													
NA (N, %)	0.026	0 (0.0)	0 (0.0)	0 (0.0)	0 (0.0)	0 (0.0)	0 (0.0)	0 (0.0)	0 (0.0)	0 (0.0)	0 (0.0)	0 (0.0)	0 (0.0)
median (IQR)		59.0 (54.0-66.0)	60.0 (55.0-66.0)	60.9 (53.3-67.0)	62.0 (54.4-67.3)	62.0 (56.0-70.0)	65.0 (58.0-70.8)	60.1 (54.3-66.7)	58.8 (53.4-66.0)	62.0 (56.0-68.8)	60.0 (52.7-66.0)	58.8 (56.0-67.8)	66.7 (61.2-72.4)
Follow-up time (years)													
NA (N, %)		0 (0.0)	0 (0.0)	0 (0.0)	0 (0.0)	0 (0.0)	0 (0.0)	0 (0.0)	1 (2.2)	0 (0.0)	0 (0.0)	0 (0.0)	0 (0.0)
median (IQR)		5.1 (3.4-7.9)	5.4 (4.0-7.9)	3.9 (3.1-5.1)	4.0 (2.0-5.1)	3.9 (2.2-5.0)	4.3 (3.2-6.2)	3.0 (1.2-4.9)	4.3 (2.3-5.2)	4.7 (2.2-6.2)	5.1 (3.1-6.5)	5.0 (3.6-5.6)	4.1 (3.0-5.0)
Primary Site Group (N, %)													
NA		0 (0.0)	0 (0.0)	0 (0.0)	0 (0.0)	0 (0.0)	0 (0.0)	0 (0.0)	0 (0.0)	0 (0.0)	0 (0.0)	0 (0.0)	0 (0.0)
OPC		234 (80.4)	114 (72.6)	104 (56.5)	52 (56.5)	68 (54.8)	15 (35.7)	52 (57.8)	25 (55.6)	47 (54.7)	23 (53.5)	20 (58.8)	10 (58.8)
OCC		48 (16.5)	34 (21.7)	56 (30.4)	28 (30.4)	56 (45.2)	27 (64.3)	36 (40.0)	19 (42.2)	20 (23.3)	10 (23.3)	12 (35.3)	6 (35.3)
Larynx/ hypopharynx		9 (3.1)	5 (3.2)	6 (3.3)	3 (3.3)	0 (0.0)	0 (0.0)	2 (2.2)	1 (2.2)	15 (17.4)	8 (18.6)	2 (5.9)	1 (5.9)
Other		0 (0.0)	4 (2.5)	18 (9.8)	9 (9.8)	0 (0.0)	0 (0.0)	0 (0.0)	0 (0.0)	4 (4.7)	2 (4.7)	0 (0.0)	0 (0.0)
T stage (N, %)													
NA		0 (0.0)	0 (0.0)	0 (0.0)	1 (1.1)	0 (0.0)	0 (0.0)	0 (0.0)	0 (0.0)	0 (0.0)	0 (0.0)	0 (0.0)	1 (5.9)
Tx		3 (1.0)	0 (0.0)	1 (0.5)	0 (0.0)	0 (0.0)	0 (0.0)	0 (0.0)	0 (0.0)	0 (0.0)	0 (0.0)	0 (0.0)	0 (0.0)
T0		4 (1.4)	1 (0.6)	9 (4.9)	4 (4.3)	0 (0.0)	0 (0.0)	0 (0.0)	0 (0.0)	0 (0.0)	0 (0.0)	0 (0.0)	0 (0.0)
T1		62 (21.3)	20 (12.7)	23 (12.5)	12 (13.0)	28 (22.6)	4 (9.5)	23 (25.6)	11 (24.4)	8 (9.3)	5 (11.6)	2 (5.9)	0 (0.0)
T1c		0 (0.0)	0 (0.0)	0 (0.0)	0 (0.0)	0 (0.0)	0 (0.0)	0 (0.0)	0 (0.0)	0 (0.0)	0 (0.0)	1 (2.9)	0 (0.0)
T2		100 (34.4)	51 (32.5)	62 (33.7)	28 (30.4)	28 (22.6)	21 (50.0)	39 (43.3)	19 (42.2)	27 (31.4)	12 (27.9)	5 (14.7)	2 (11.8)
T2b		1 (0.3)	0 (0.0)	0 (0.0)	0 (0.0)	0 (0.0)	0 (0.0)	0 (0.0)	0 (0.0)	0 (0.0)	0 (0.0)	0 (0.0)	0 (0.0)
T3		76 (26.1)	33 (21.0)	28 (15.2)	9 (9.8)	15 (12.1)	9 (21.4)	12 (13.3)	10 (22.2)	12 (14.0)	5 (11.6)	7 (20.6)	2 (11.8)
T4		39 (13.4)	50 (31.8)	4 (2.2)	4 (4.3)	0 (0.0)	0 (0.0)	16 (17.8)	5 (11.1)	39 (45.3)	21 (48.8)	6 (17.6)	6 (35.3)
T4a		6 (2.1)	2 (1.3)	56 (30.4)	30 (32.6)	49 (39.5)	8 (19.0)	0 (0.0)	0 (0.0)	0 (0.0)	0 (0.0)	7 (20.6)	3 (17.6)
T4b		0 (0.0)	0 (0.0)	1 (0.5)	4 (4.3)	4 (3.2)	0 (0.0)	0 (0.0)	0 (0.0)	0 (0.0)	0 (0.0)	6 (17.6)	3 (17.6)
N stage (N, %)													
NA		0 (0.0)	0 (0.0)	2 (1.1)	1 (1.1)	0 (0.0)	0 (0.0)	0 (0.0)	0 (0.0)	0 (0.0)	0 (0.0)	0 (0.0)	1 (5.9)
N0		39 (13.4)	21 (13.4)	60 (32.6)	25 (27.2)	54 (43.5)	15 (35.7)	24 (26.7)	17 (37.8)	21 (24.4)	7 (16.3)	11 (32.4)	3 (17.6)
Nx		1 (0.3)	0 (0.0)	1 (0.5)	0 (0.0)	0 (0.0)	0 (0.0)	0 (0.0)	0 (0.0)	0 (0.0)	0 (0.0)	0 (0.0)	0 (0.0)
N1		36 (12.4)	16 (10.2)	21 (11.4)	8 (8.7)	19 (15.3)	5 (11.9)	19 (21.1)	10 (22.2)	12 (14.0)	6 (14.0)	14 (41.2)	3 (17.6)

N2		86 (29.6)	63 (40.1)	0 (0.0)	0 (0.0)	0 (0.0)	0 (0.0)	3 (3.3)	0 (0.0)	0 (0.0)	1 (2.3)	0 (0.0)	1 (5.9)
N2a		10 (3.4)	3 (1.9)	15 (8.2)	3 (3.3)	3 (2.4)	2 (4.8)	2 (2.2)	1 (2.2)	7 (8.1)	1 (2.3)	1 (2.9)	5 (29.4)
N2b		75 (25.8)	30 (19.1)	67 (36.4)	38 (41.3)	31 (25.0)	16 (38.1)	25 (27.8)	11 (24.4)	25 (29.1)	15 (34.9)	14 (41.2)	3 (17.6)
N2c		37 (12.7)	21 (13.4)	13 (7.1)	13 (14.1)	14 (11.3)	3 (7.1)	15 (16.7)	5 (11.1)	18 (20.9)	11 (25.6)	4 (11.8)	0 (0.0)
N3		7 (2.4)	3 (1.9)	5 (2.7)	4 (4.3)	3 (2.4)	1 (2.4)	2 (2.2)	1 (2.2)	3 (3.5)	2 (4.7)	0 (0.0)	1 (5.9)
M stage (N, %)													
NA		291 (100.0)	157 (100.0)	11 (6.0)	2 (2.2)	21 (16.9)	0 (0.0)	0 (0.0)	0 (0.0)	0 (0.0)	0 (0.0)	0 (0.0)	1 (5.9)
M0		NA	NA	172 (93.5)	90 (97.8)	103 (83.1)	42 (100.0)	88 (97.8)	45 (100.0)	86 (100.0)	43 (100.0)	34 (100.0)	16 (94.1)
M1				1 (0.5)	0 (0.0)	0 (0.0)	0 (0.0)	2 (2.2)	0 (0.0)	0 (0.0)	0 (0.0)	0 (0.0)	0 (0.0)
Mandible volume (cc)*													
NA (N, %)	<0.001	0 (0.0)	0 (0.0)	1 (0.5)	2 (2.2)	0 (0.0)	0 (0.0)	0 (0.0)	1 (2.2)	0 (0.0)	0 (0.0)	0 (0.0)	0 (0.0)
median (IQR)		85.7 (75.4-97.3)	86.5 (75.6-97.5)	55.1 (47.9-64.6)	62.5 (48.2-74.6)	71.8 (61.2-80.3)	72.4 (60.5-88.3)	67.4 (58.2-73.0)	70.5 (62.3-81.5)	56.1 (47.5-62.7)	54.9 (46.8-65.4)	67.1 (52.8-73.0)	66.5 (56.3-72.3)
HPV (N, %)													
NA		291 (100.0)	157 (100.0)	117 (63.6)	65 (70.7)	101 (81.5)	38 (90.5)	90 (100.0)	45 (100.0)	41 (47.7)	17 (39.5)	34 (100.0)	17 (100.0)
HPV-				11 (6.0)	7 (7.6)	9 (7.3)	3 (7.1)			26 (30.2)	20 (46.5)		
HPV+				56 (30.4)	20 (21.7)	14 (11.3)	1 (2.4)			19 (22.1)	6 (14.0)		
Smoking status (N, %)*													
NA	<0.001	0 (0.0)	0 (0.0)	1 (0.5)	0 (0.0)	0 (0.0)	0 (0.0)	0 (0.0)	0 (0.0)	0 (0.0)	0 (0.0)	1 (2.9)	0 (0.0)
Current		44 (15.1)	27 (17.2)	50 (27.2)	47 (51.1)	54 (43.5)	22 (52.4)	49 (54.4)	32 (71.1)	37 (43.0)	29 (67.4)	18 (52.9)	12 (70.6)
Former		110 (37.8)	72 (45.9)	80 (43.5)	26 (28.3)	54 (43.5)	15 (35.7)	24 (26.7)	8 (17.8)	32 (37.2)	12 (27.9)	9 (26.5)	3 (17.6)
Never		137 (47.1)	58 (36.9)	53 (28.8)	19 (20.7)	16 (12.9)	5 (11.9)	17 (18.9)	5 (11.1)	17 (19.8)	2 (4.7)	6 (17.6)	2 (11.8)
Smoking amount (N, %)													
NA		0 (0.0)	0 (0.0)	38 (20.7)	19 (20.7)	119 (96.0)	38 (90.5)	90 (100.0)	45 (100.0)	13 (15.1)	15 (34.9)	34 (100.0)	17 (100.0)
> 10 pack years		112 (38.5)	69 (43.9)	73 (39.7)	45 (48.9)	0 (0.0)	0 (0.0)			46 (53.5)	22 (51.2)		
< 10 pack years		42 (14.4)	30 (19.1)	19 (10.3)	5 (5.4)	0 (0.0)	0 (0.0)	NA	NA	27 (31.4)	6 (14.0)	NA	NA
None		137 (47.1)	58 (36.9)	53 (28.8)	19 (20.7)	16 (12.9)	5 (11.9)			17 (19.8)	2 (4.7)		
Tobacco pack years													
NA (N, %)		0 (0.0)	0 (0.0)	184 (100.0)	92 (100.0)	124 (100.0)	42 (100.0)	90 (100.0)	45 (100.0)	14 (16.3)	15 (34.9)	34 (100.0)	17 (100.0)
median (IQR)		1.0 (0.0-30.0)	6.0 (0.0-30.0)	NA	NA	NA	NA	NA	NA	25.0 (0.0-41.3)	30.0 (16.5-40.0)	NA	NA
Alcohol status (N, %)													
NA		291 (100.0)	157 (100.0)	1 (0.5)	0 (0.0)	0 (0.0)	0 (0.0)	90 (100.0)	45 (100.0)	2 (2.3)	3 (7.0)	1 (2.9)	0 (0.0)
Current				137 (74.5)	71 (77.2)	77 (62.1)	29 (69.0)			64 (74.4)	35 (81.4)	16 (47.1)	10 (58.8)
Former		NA	NA	25 (13.6)	8 (8.7)	18 (14.5)	3 (7.1)	NA	NA	6 (7.0)	1 (2.3)	1 (2.9)	3 (17.6)
Never				21 (11.4)	13 (14.1)	29 (23.4)	10 (23.8)			14 (16.3)	4 (9.3)	16 (47.1)	4 (23.5)
Alcohol amount (N, %)													
NA		291 (100.0)	157 (100.0)	11 (6.0)	8 (8.7)	97 (78.2)	33 (78.6)	90 (100.0)	45 (100.0)	86 (100.0)	43 (100.0)	34 (100.0)	17 (100.0)
> 21 units week				47 (25.5)	32 (34.8)	0 (0.0)	0 (0.0)						

< 21 units week		NA	NA	94 (51.1)	31 (33.7)	0 (0.0)	0 (0.0)	NA	NA	NA	NA	NA	NA
None				32 (17.4)	21 (22.8)	27 (21.8)	9 (21.4)						
Pre-RT extractions (N, %)*													
NA	<0.001	0 (0.0)	0 (0.0)	11 (6.0)	0 (0.0)	0 (0.0)	0 (0.0)	0 (0.0)	0 (0.0)	0 (0.0)	0 (0.0)	0 (0.0)	0 (0.0)
Yes		206 (70.8)	64 (40.8)	113 (61.4)	55 (59.8)	48 (38.7)	16 (38.1)	63 (70.0)	36 (80.0)	37 (43.0)	31 (72.1)	1 (2.9)	2 (11.8)
No		85 (29.2)	93 (59.2)	60 (32.6)	37 (40.2)	76 (61.3)	26 (61.9)	27 (30.0)	9 (20.0)	49 (57.0)	12 (27.9)	33 (97.1)	15 (88.2)
Time extraction to RT (days)													
NA (N, %)		291 (100.0)	157 (100.0)	107 (58.2)	92 (100.0)	76 (61.3)	26 (61.9)	90 (100.0)	45 (100.0)	49 (57.0)	12 (27.9)	34 (100.0)	17 (100.0)
median (IQR)		NA	NA	38.0 (25.0-56.0)	NA	32.0 (23.3-42.3)	29.0 (18.3-40.3)	NA	NA	20.0 (14.0-31.0)	20.0 (18.0-26.5)	NA	NA
Chemotherapy (N, %)*													
NA	<0.001	0 (0.0)	4 (2.5)	1 (0.5)	0 (0.0)	0 (0.0)	0 (0.0)	0 (0.0)	0 (0.0)	0 (0.0)	0 (0.0)	0 (0.0)	0 (0.0)
Yes		250 (85.9)	138 (87.9)	110 (59.8)	59 (64.1)	51 (41.1)	14 (33.3)	25 (27.8)	13 (28.9)	63 (73.3)	39 (90.7)	27 (79.4)	15 (88.2)
No		41 (14.1)	15 (9.6)	73 (39.7)	33 (35.9)	73 (58.9)	28 (66.7)	65 (72.2)	32 (71.1)	23 (26.7)	4 (9.3)	7 (20.6)	2 (11.8)
RT technique (N, %)*	<0.001												
NA		0 (0.0)	0 (0.0)	0 (0.0)	0 (0.0)	0 (0.0)	0 (0.0)	0 (0.0)	0 (0.0)	0 (0.0)	0 (0.0)	0 (0.0)	0 (0.0)
IMRT		262 (90.0)	143 (91.1)	99 (53.8)	53 (57.6)	124 (100.0)	42 (100.0)	46 (51.1)	25 (55.6)	78 (90.7)	40 (93.0)	0 (0.0)	0 (0.0)
VMAT		29 (10.0)	14 (8.9)	85 (46.2)	39 (42.4)	0 (0.0)	0 (0.0)	44 (48.9)	20 (44.4)	8 (9.3)	3 (7.0)	34 (100.0)	17 (100.0)
Primary RT/PORT (N, %)*	<0.001												
NA		0 (0.0)	0 (0.0)	0 (0.0)	0 (0.0)	0 (0.0)	0 (0.0)	0 (0.0)	0 (0.0)	0 (0.0)	0 (0.0)	0 (0.0)	0 (0.0)
Primary RT		258 (88.7)	127 (80.9)	111 (60.3)	57 (62.0)	68 (54.8)	15 (35.7)	59 (65.6)	28 (62.2)	65 (75.6)	37 (86.0)	26 (76.5)	15 (88.2)
PORT		33 (11.3)	30 (19.1)	73 (39.7)	35 (38.0)	56 (45.2)	27 (64.3)	31 (34.4)	17 (37.8)	21 (24.4)	6 (14.0)	8 (23.5)	2 (11.8)
Fraction size (N, %)*													
NA		0 (0.0)	0 (0.0)	0 (0.0)	0 (0.0)	0 (0.0)	0 (0.0)	0 (0.0)	0 (0.0)	0 (0.0)	0 (0.0)	0 (0.0)	0 (0.0)
median	<0.001	2.1	2.1	2.2	2.2	2.0	2.0	2.0	2.0	2.0	2.0	2.1	2.1
range		2.0-2.1	2.0-2.1	2.0-2.8	2.0-2.8	2.0-2.0	2.0-2.0	1.2-2.1	1.4-2.1	1.5-2.0	2.0-2.0	1.8-2.1	2.0-2.1
> 2Gy/fraction		258 (88.7)	127 (80.9)	121 (65.8)	62 (67.4)	0 (0.0)	0 (0.0)	12 (13.3)	8 (17.8)	1 (1.2)	0 (0.0)	24 (70.6)	15 (88.2)
<= 2Gy/fraction		33 (11.3)	30 (19.1)	63 (34.2)	30 (32.6)	124 (100.0)	42 (100.0)	78 (86.7)	37 (82.2)	85 (98.8)	43 (100.0)	10 (29.4)	2 (11.8)
ECOG performance (N, %)													
NA		291 (100.0)	157 (100.0)	45 (24.5)	20 (21.7)	56 (45.2)	27 (64.3)	0 (0.0)	0 (0.0)	2 (2.3)	1 (2.3)	25 (73.5)	11 (64.7)
0		NA	NA	86 (46.7)	41 (44.6)	18 (14.5)	4 (9.5)	39 (43.3)	22 (48.9)	40 (46.5)	9 (20.9)	5 (14.7)	3 (17.6)
1				46 (25.0)	30 (32.6)	48 (38.7)	11 (26.2)	33 (36.7)	17 (37.8)	21 (24.4)	6 (14.0)	4 (11.8)	3 (17.6)
2				7 (3.8)	1 (1.1)	2 (1.6)	0 (0.0)	16 (17.8)	5 (11.1)	22 (25.6)	27 (62.8)	0 (0.0)	0 (0.0)
3				0 (0.0)	0 (0.0)	0 (0.0)	0 (0.0)	2 (2.2)	1 (2.2)	1 (1.2)	0 (0.0)	0 (0.0)	0 (0.0)
4				0 (0.0)	0 (0.0)	0 (0.0)	0 (0.0)	0 (0.0)	0 (0.0)	0 (0.0)	0 (0.0)	0 (0.0)	0 (0.0)
5				0 (0.0)	0 (0.0)	0 (0.0)	0 (0.0)	0 (0.0)	0 (0.0)	0 (0.0)	0 (0.0)	0 (0.0)	0 (0.0)
Xerostomia baseline (N, %)													

NA		291 (100.0)	157 (100.0)	55 (29.9)	33 (35.9)	124 (100.0)	42 (100.0)	90 (100.0)	45 (100.0)	86 (100.0)	43 (100.0)	3 (8.8)	0 (0.0)
0		NA	NA	106 (57.6)	53 (57.6)	NA	NA	NA	NA	NA	NA	31 (91.2)	17 (100.0)
1				19 (10.3)	5 (5.4)							0 (0.0)	0 (0.0)
2				0 (0.0)	1 (1.1)							0 (0.0)	0 (0.0)
3				0 (0.0)	0 (0.0)							0 (0.0)	0 (0.0)
4				4 (2.2)	0 (0.0)							0 (0.0)	0 (0.0)
Xerostomia 1-year post-RT (N, %)													
NA		291 (100.0)	157 (100.0)	7 (3.8)	8 (8.7)	124 (100.0)	42 (100.0)	7 (7.8)	3 (6.7)	86 (100.0)	43 (100.0)	3 (8.8)	1 (5.9)
0		NA	NA	1 (0.5)	3 (3.3)	NA	NA	21 (23.3)	0 (0.0)	NA	NA	0 (0.0)	0 (0.0)
1				30 (16.3)	24 (26.1)			33 (36.7)	11 (24.4)			20 (58.8)	1 (5.9)
2				146 (79.3)	56 (60.9)			25 (27.8)	22 (48.9)			10 (29.4)	7 (41.2)
3				0 (0.0)	1 (1.1)			4 (4.4)	9 (20.0)			1 (2.9)	6 (35.3)
4				0 (0.0)	0 (0.0)			0 (0.0)	0 (0.0)			0 (0.0)	2 (11.8)
Dmean parotids (Gy)													
NA (N, %)		291 (100.0)	157 (100.0)	184 (100.0)	92 (100.0)	0 (0.0)	0 (0.0)	90 (100.0)	45 (100.0)	86 (100.0)	43 (100.0)	1 (2.9)	0 (0.0)
median (IQR)		NA	NA	NA	NA	24.4 (15.8-34.1)	22.8 (11.5-33.4)	NA	NA	NA	NA	26.3 (22.1-32.3)	24.2 (22.1-33.0)
Time to ORN (years)													
NA (N, %)		n/a	0 (0.0)	n/a	0 (0.0)	n/a	0 (0.0)	n/a	0 (0.0)	n/a	11 (25.6)	n/a	0 (0.0)
median (IQR)			7.4 (4.2-10.0)		1.1 (0.5-2.2)		1.0 (0.6-2.4)		0.8 (0.4-1.6)		0.6 (0.3-1.3)		1.2 (0.4-2.2)
ORN grade Notani (N, %)													
NA		n/a	NA	n/a	0 (0.0)	n/a	1 (2.4)	n/a	0 (0.0)	n/a	NA	n/a	0 (0.0)
1					37 (40.2)		14 (33.3)		19 (42.2)				3 (17.6)
2					12 (13.0)		11 (26.2)		18 (40.0)				9 (52.9)
3					43 (46.7)		16 (38.1)		8 (17.8)				5 (29.4)
ORN grade CTCAE (N, %)													
NA		n/a	NA	n/a	NA	n/a	0 (0.0)	n/a	0 (0.0)	n/a	0 (0.0)	n/a	0 (0.0)
1							2 (4.8)		11 (24.4)		2 (4.7)		1 (5.9)
2							17 (40.5)		22 (48.9)		9 (20.9)		10 (58.8)
3							23 (54.8)		12 (26.7)		32 (74.4)		6 (35.3)
ORN grade Tsai (N, %)													
NA		n/a	0 (0.0)	n/a	NA	n/a	NA	n/a	NA	n/a	0 (0.0)	n/a	NA
1			21 (13.4)								3 (7.0)		
2			33 (21.0)								18 (41.9)		
3			47 (29.9)								13 (30.2)		
4			56 (35.7)								9 (20.9)		

Table B3. Notani and Tsai ORN grading systems.

**Notani ORN Grading System**	
**Stage I**	ORN confined to the alveolar bone, which is the part of the jawbone that supports the teeth.
**Stage II**	ORN extending to the mandibular bone, but without pathological fractures.
**Stage III**	ORN with extensive bone involvement, possibly including pathological fractures, or ORN extending beyond the mandibular body (e.g., to the ramus of the mandible or the skull base).
**Tsai ORN Grading System**	
**Stage 1**	Bone exposure or radiographic evidence of bone necrosis without symptoms or infection.
**Stage 2**	Bone exposure with symptoms such as pain, and possibly secondary infection, but without a pathologic fracture or fistula.
**Stage 3**	More severe involvement with complications such as pathological fracture, fistula, or involvement of adjacent structures (e.g., skin or muscles).

Supplement C. Univariable analyses

Table C1: Univariable analysis on the matched datasets (1,263 HNC patients, 415 ORN cases) for the generic cohort (including all tumour sites), and the two sub-cohorts considered, the OPC/advanced (T3/T4) larynx/hypopharynx cancer cases sub-cohort and OCC cases sub-cohort.

	**Generic cohort (all primary tumour sites)**		**OPC/larynx/hypopharynx sub-cohort**		**OCC sub-cohort**	
**Variable**	**p_LRT**	**OR (95% CI)**	**p_LRT**	**OR (95% CI)**	**p_LRT**	**OR (95% CI)**
Sex	0.683	0.94 (0.72-1.25)	0.080	0.70 (0.47-1.04)	0.845	1.05 (0.67-1.64)
Age	0.814	1.00 (0.99, 1.01)	0.770	1.00 (0.99-1.02)	0.798	1.00 (0.98-1.02)
Mandible volume	0.013	1.01 (1.00, 1.01)	0.079	1.01 (1.00-1.02)	0.024	1.01 (1.00-1.02)
Smoking status	0.002	1.62 (1.26, 2.08)	0.003	1.61 (1.18-2.20)	0.004	1.95 (1.24-3.06)
Pack Years	0.094	1.01 (1.00, 1.01)	0.475	1.21 (0.72-2.04)	0.091	2.13 (0.89-5.10)
Xerostomia 1 year	0.015	0.61 (0.41, 0.91)	0.132	0.67 (0.40-1.13)	0.202	0.64 (0.32-1.27)
Pre-RT extraction	0.026	1.31 (1.03, 1.66)	0.003	1.58 (1.17-2.14)	0.550	0.88 (0.57-1.35)
Time extraction to RT	0.002	0.97 (0.95, 0.99)	0.090	2.34 (0.88-6.23)	0.203	2.64 (0.59-11.75)
Chemotherapy	0.042	1.31 (1.01, 1.69)	0.120	1.36 (0.92-2.01)	0.072	1.51 (0.96-2.37)
PORT/PrimaryRT	0.504	1.09 (0.84, 1.42)	0.425	0.68 (0.27-1.74)	0.826	1.06 (0.62-1.83)
RT technique	0.753	1.04 (0.80, 1.36)	0.643	1.09 (0.76-1.55)	0.947	1.02 (0.64-1.61)
Fraction size	0.402	1.11 (0.87, 1.40)	0.072	1.35 (0.97-1.87)	0.799	1.08 (0.60-1.92)
V05Gy	0.310	1.01 (0.99, 1.02)	0.173	1.02 (0.99-1.06)	0.406	1.01 (0.99-1.03)
V10Gy	0.291	1.01 (0.99, 1.02)	0.084	1.02 (1.00-1.04)	0.553	1.01 (0.99-1.02)
V15Gy	0.055	1.01 (1.00, 1.02)	0.004	1.03 (1.01-1.05)	0.449	1.01 (0.99-1.02)
V20Gy	0.001	1.01 (1.01, 1.02)	<0.001	1.04 (1.02-1.05)	0.256	1.01 (0.99-1.02)
V25Gy	<0.001	1.02 (1.01, 1.02)	<0.001	1.03 (1.02-1.05)	0.201	1.01 (1.00-1.02)
V30Gy	<0.001	1.02 (1.01, 1.03)	<0.001	1.03 (1.02-1.04)	0.080	1.01 (1.00-1.02)
V35Gy	<0.001	1.02 (1.01, 1.03)	<0.001	1.03 (1.02-1.03)	0.034	1.01 (1.00-1.02)
V40Gy	<0.001	1.02 (1.01, 1.03)	<0.001	1.03 (1.02-1.03)	0.023	1.01 (1.00-1.02)
V45Gy	<0.001	1.02 (1.02, 1.03)	<0.001	1.03 (1.02-1.04)	0.011	1.01 (1.00-1.03)
V50Gy	<0.001	1.02 (1.02, 1.03)	<0.001	1.03 (1.02-1.04)	0.006	1.01 (1.00-1.03)
V55Gy	<0.001	1.03 (1.02, 1.03)	<0.001	1.03 (1.02-1.04)	<0.001	1.02 (1.01-1.03)
V60Gy	<0.001	1.03 (1.02, 1.04)	<0.001	1.04 (1.03-1.05)	<0.001	1.02 (1.01-1.03)
V65Gy	<0.001	1.03 (1.02, 1.04)	<0.001	1.04 (1.03-1.06)	0.062	1.01 (1.00-1.03)
V70Gy	<0.001	1.05 (1.03, 1.07)	<0.001	1.10 (1.07-1.13)	0.257	1.01 (1.00-1.03)
D02%	<0.001	1.05 (1.03, 1.07)	<0.001	1.06 (1.03-1.10)	0.011	1.05 (1.01-1.10)
D05%	<0.001	1.06 (1.04, 1.08)	<0.001	1.08 (1.04-1.11)	0.003	1.06 (1.02-1.11)
D10%	<0.001	1.07 (1.05, 1.09)	<0.001	1.08 (1.05-1.11)	0.001	1.07 (1.03-1.11)
D15%	<0.001	1.07 (1.05, 1.09)	<0.001	1.08 (1.05-1.11)	0.001	1.07 (1.03-1.11)
D20%	<0.001	1.07 (1.05, 1.09)	<0.001	1.08 (1.06-1.11)	<0.001	1.07 (1.03-1.11)
D25%	<0.001	1.071.05, 1.08)	<0.001	1.08 (1.05-1.10)	<0.001	1.06 (1.03-1.10)
D30%	<0.001	1.06 (1.05, 1.08)	<0.001	1.07 (1.05-1.09)	<0.001	1.06 (1.02-1.09)
D35%	<0.001	1.05 (1.04, 1.06)	<0.001	1.06 (1.05-1.08)	<0.001	1.05 (1.02-1.08)
D40%	<0.001	1.05 (1.04, 1.06)	<0.001	1.06 (1.04-1.08)	<0.001	1.05 (1.02-1.07)
D45%	<0.001	1.05 (1.04, 1.06)	<0.001	1.06 (1.04-1.08)	<0.001	1.04 (1.02-1.06)
D50%	<0.001	1.04 (1.03, 1.05)	<0.001	1.06 (1.04-1.07)	<0.001	1.03 (1.01-1.05)
D55%	<0.001	1.04 (1.03, 1.05)	<0.001	1.05 (1.05-1.07)	0.003	1.02 (1.01-1.04)
D60%	<0.001	1.03 (1.02, 1.04)	<0.001	1.05 (1.04-1.07)	0.021	1.02 (1.00-1.03)
D65%	<0.001	1.03 (1.02, 1.04)	<0.001	1.05 (1.03-1.07)	0.095	1.01 (1.00-1.03)
D70%	<0.001	1.03 (1.02, 1.04)	<0.001	1.05 (1.03-1.06)	0.219	1.01 (1.00-1.02)
D75%	<0.001	1.02 (1.01, 1.03)	<0.001	1.05 (1.03-1.06)	0.501	1.00 (0.99-1.02)
D80%	<0.001	1.02 (1.01, 1.03)	<0.001	1.05 (1.03-1.07)	0.869	1.00 (0.99-1.01)
D85%	<0.001	1.02 (1.01, 1.03)	<0.001	1.04 (1.03-1.06)	0.959	1.00 (0.99-1.01)
D90%	0.006	1.01 (1.00, 1.03)	<0.001	1.04 (1.02-1.05)	0.814	1.00 (0.98-1.01)
D95%	0.016	1.02 (1.00, 1.03)	<0.001	1.04 (1.02-1.06)	0.807	1.00 (0.97-1.02)
D98%	0.040	1.02 (1.00, 1.03)	0.001	1.04 (1.02-1.06)	0.837	1.00 (0.96-1.03)

Supplement D. Mandible volume analyses

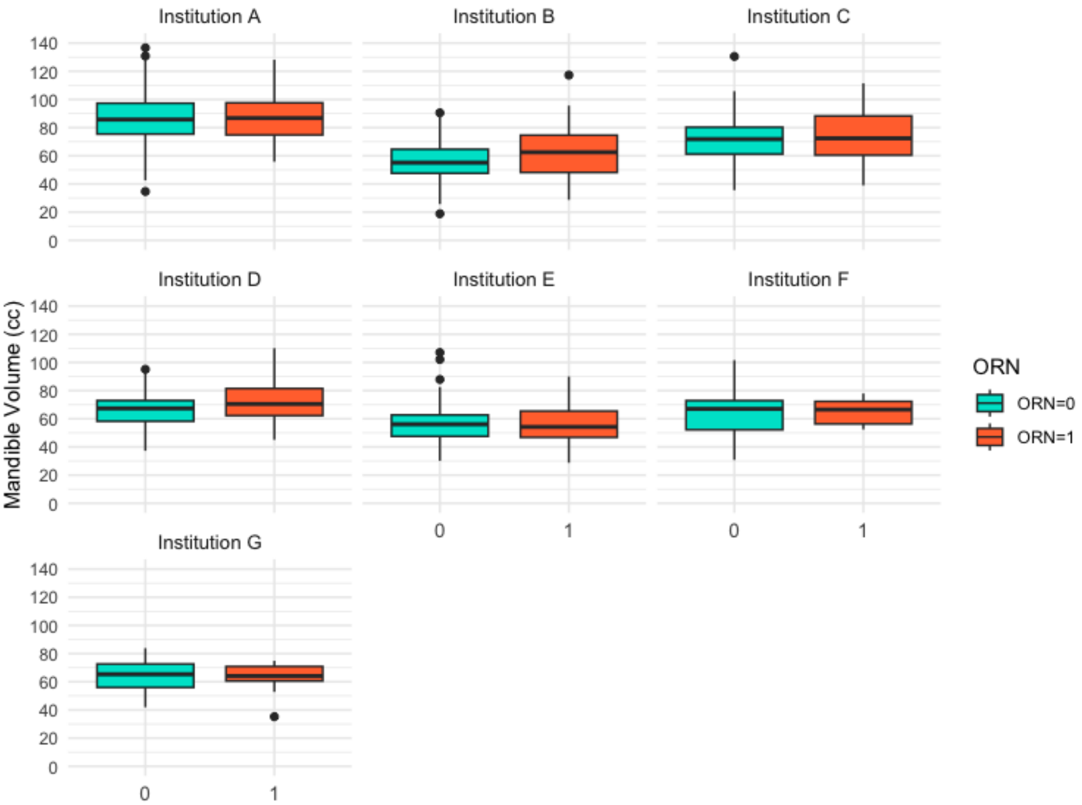

Figure D1. Institution-specific distribution of mandible volumes by group (ORN vs. control) across institutions A to G (data from institution H was not directly available for aggregation in this plot).

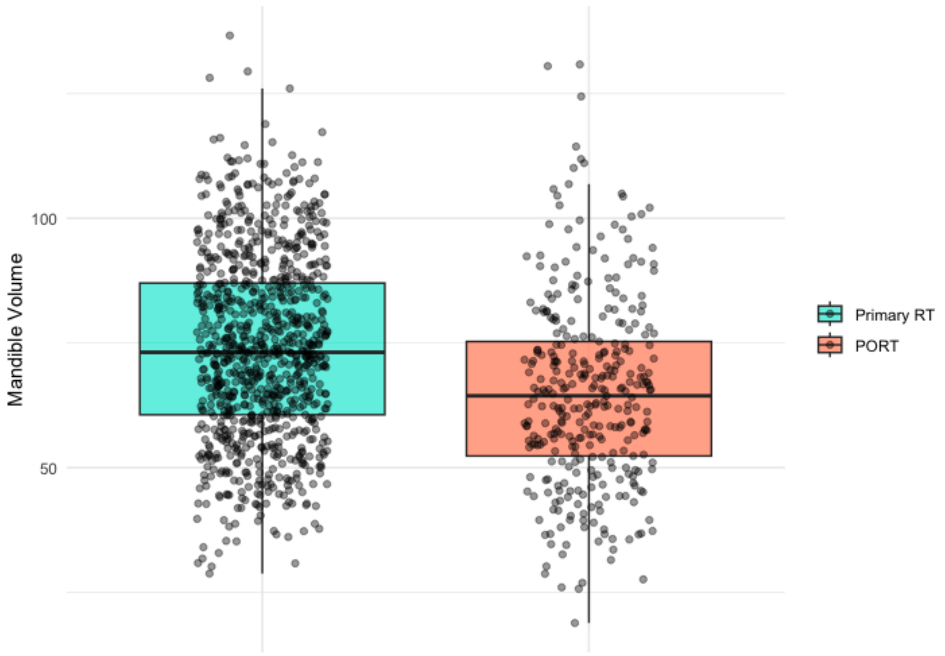

Figure D2. Distribution of mandible volume values by PORT status across institutions A to G (data from institution H was not directly available for aggregation in this plot). Smaller mandible volumes observed in patients who underwent surgery prior RT (i.e., PORT positive status) (median 64.4 cc, IQR 52.3-75.3) compared to patients receiving RT as their primary treatment (73.1 cc, 60.6-87.0).

Supplement E. Dosimetric data

Exact prescription dose and fractionation data were not uniformly available for all patients; however, a summary of the schedules employed across participating centres has been compiled and is presented below:

Institution A: Generally, the prescribed dose to primary tumour range was 68 to 72 Gy for definitive treatment (typically, 2.12 Gy in 33 fractions 5 times per week), 60 to 66 Gy for postoperative indications (typically, 2 Gy in 30-33 fractions 5 times per week), and 57 Gy to the elective lymph node levels (1.72 Gy in 33 fractions).

Institution B: Radical primary cases were prescribed a total dose of 65–70 Gy in 30–35 fractions and 55 Gy in 20 fractions in selected cases. Radical post-operative (PORT) cases were prescribed 60–66 Gy in 30–33 fractions and 50 Gy in 20 fractions in selected cases.

Institution C: Radical primary cases were uniformly prescribed 70 Gy in 35 fractions. Radical post-operative (PORT) cases were prescribed 66 Gy in 33 fractions or 60 Gy in 30 fractions in most cases, with a subset treated to 56 Gy in 28 fractions.

Institution D: The dose prescription was 66 or 68 Gy in 2 Gy fractions 5–6 fractions per week following the Danish Head and Neck Cancer Group (DAHANCA) national guidelines from 2013.

Institution E: Radical primary cases were most commonly prescribed 70 Gy in 35 fractions. Selected cases received 60 Gy in 30 fractions, 58 Gy in 29 fractions, or lower doses such as 54 Gy in 30 fractions and 40 Gy in 20 fractions. Radical post-operative (PORT) cases were prescribed 66 Gy in 33 fractions or 56 Gy in 28 fractions in the majority of cases, with occasional use of 50–54 Gy in 25–27 fractions.

Institution F: Radical primary cases were most commonly prescribed 70 Gy in 33 fractions, with additional regimens including 70 Gy in 35 fractions, 66 Gy in 30 fractions, and 70.2 Gy in 39 fractions in selected cases. Radical post-operative (PORT) cases were prescribed 70 Gy in 33–35 fractions or 66 Gy in 30 fractions, with alternative regimens of 62 Gy in 31 fractions, 50 Gy in 25 fractions, and 70.4 Gy in 38 fractions.

Institution G: Radical primary RT cases were predominantly prescribed 70 Gy in 35 fractions. Selected patients received 60 Gy in 30 fractions, 60 Gy in 25 fractions, 64 Gy in 32 fractions, or 83.6 Gy in 39 fractions. Radical post-operative (PORT) cases were prescribed either 70 Gy in 35 fractions or 60 Gy in 30 fractions.

Institution H: Generally, the prescribed dose to primary tumour range was 70 Gy for definitive treatment (typically, 2 Gy in 35 fractions 5 times per week), 60-66 Gy for postoperative indications (typically, 2 Gy in 30-33 fractions 5 times per week), and 54-56 Gy to the elective lymph node levels (1.86 Gy in 30 fractions).

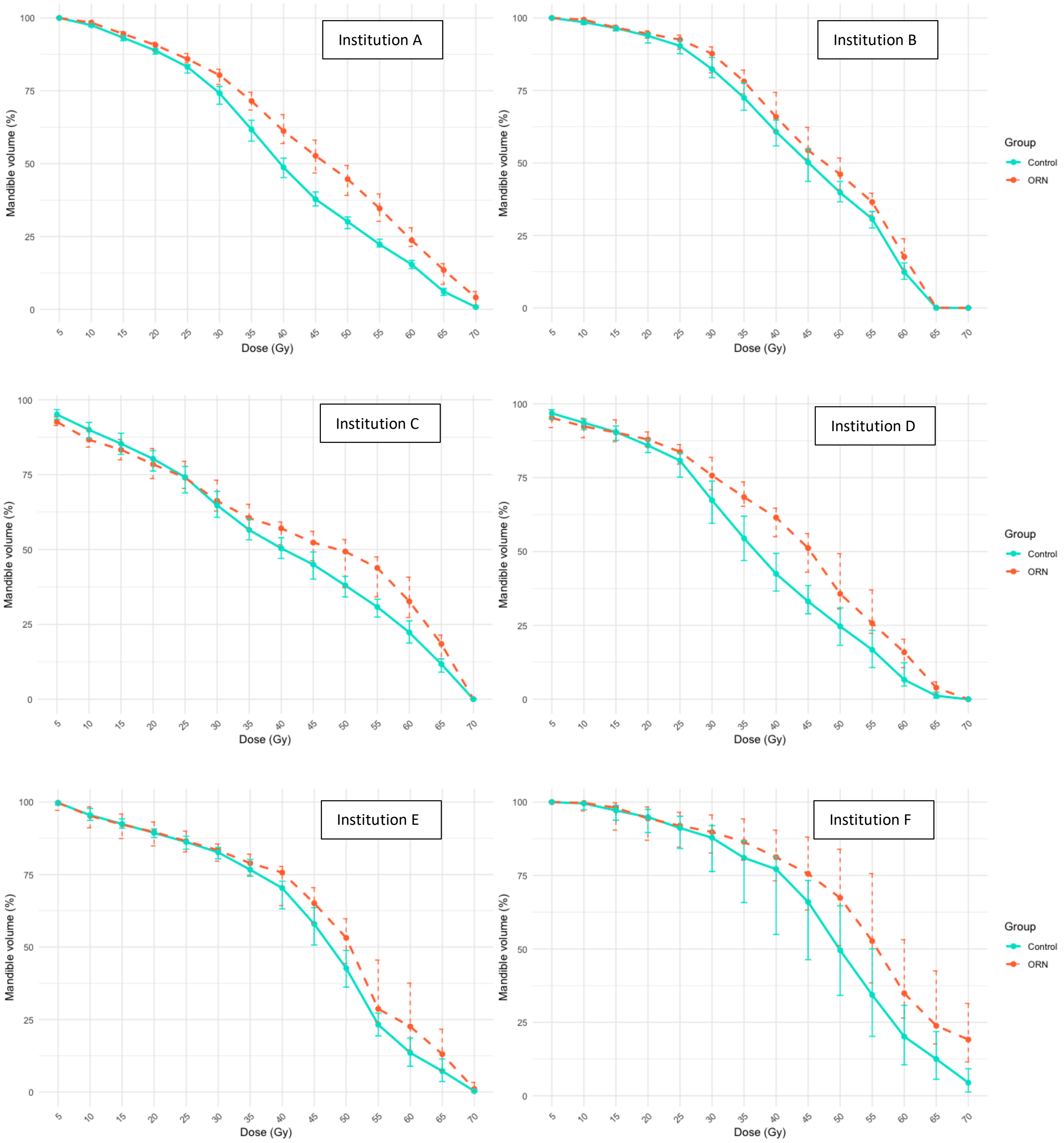
Institution A
Institution B
Institution C
Institution D
Institution E
Institution F
Mandible volume (%)
Dose (Gy)
0
25
50
75
100
5
10
15
20
25
30
35
40
45
50
55
60
65
70
Group
Control
ORN

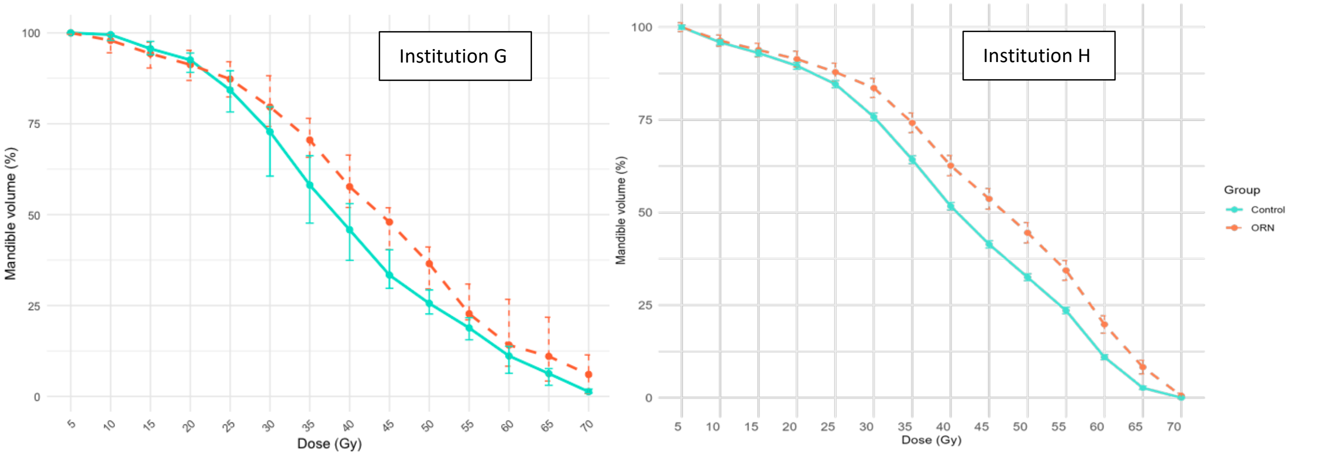

Figure E1. Institution-specific (institutions A to H) median cumulative dose-volume histogram (DVH) plots for the ORN and control groups. The medians were calculated for each DVH metric across the ORN and control groups. Error bars correspond to the 95% confidence intervals of the medians estimated using the binomial method, which identifies the lower and upper ranks corresponding to the 95% confidence bounds based on the size and distribution of the data.

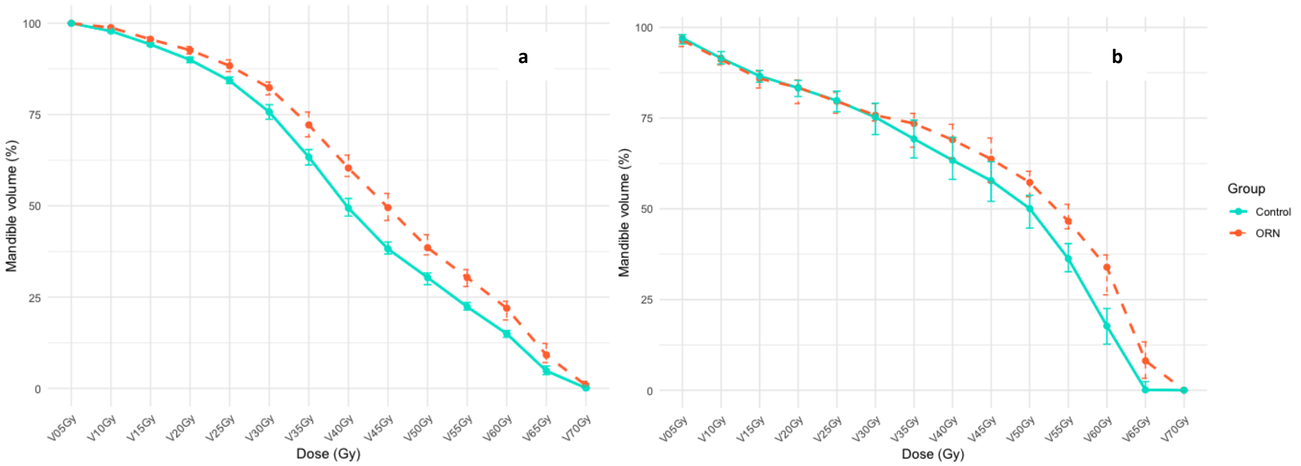

Figure E2. Median cumulative DVH plots for a) the OPC/advanced larynx/hypopharynx cancer subset and b) the OCC subset for the ORN and control groups across institutions A to G (data from institution H was not directly available for aggregation in these plots). The medians were calculated for each DVH metric across the ORN and control groups. Error bars correspond to the 95% confidence intervals of the medians estimated using the binomial method, which identifies the lower and upper ranks corresponding to the 95% confidence bounds based on the size and distribution of the data.

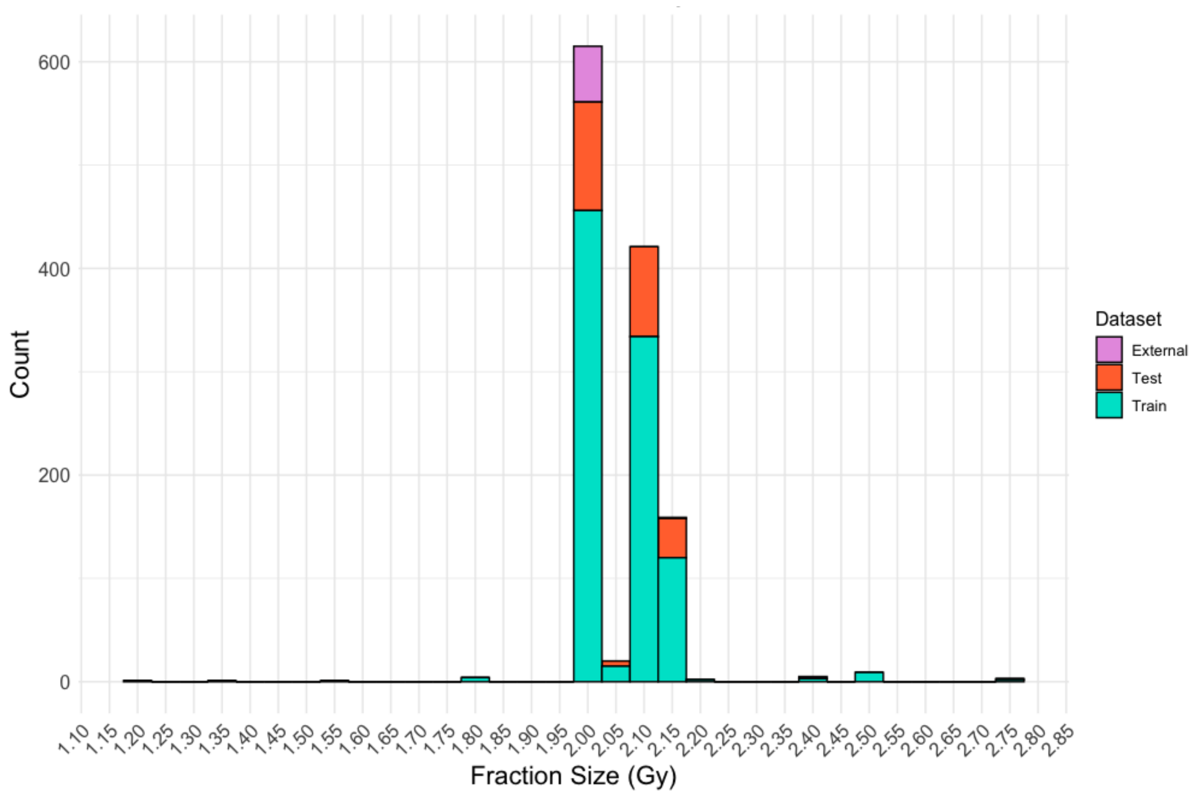

Figure E3. Distribution of fraction sizes for the train, test and external datasets across institutions A to G (data from institution H was not directly available for aggregation in this plot). Patients receiving simultaneous integrated boost plans were assigned a single fraction size corresponding to the highest dose level delivered.

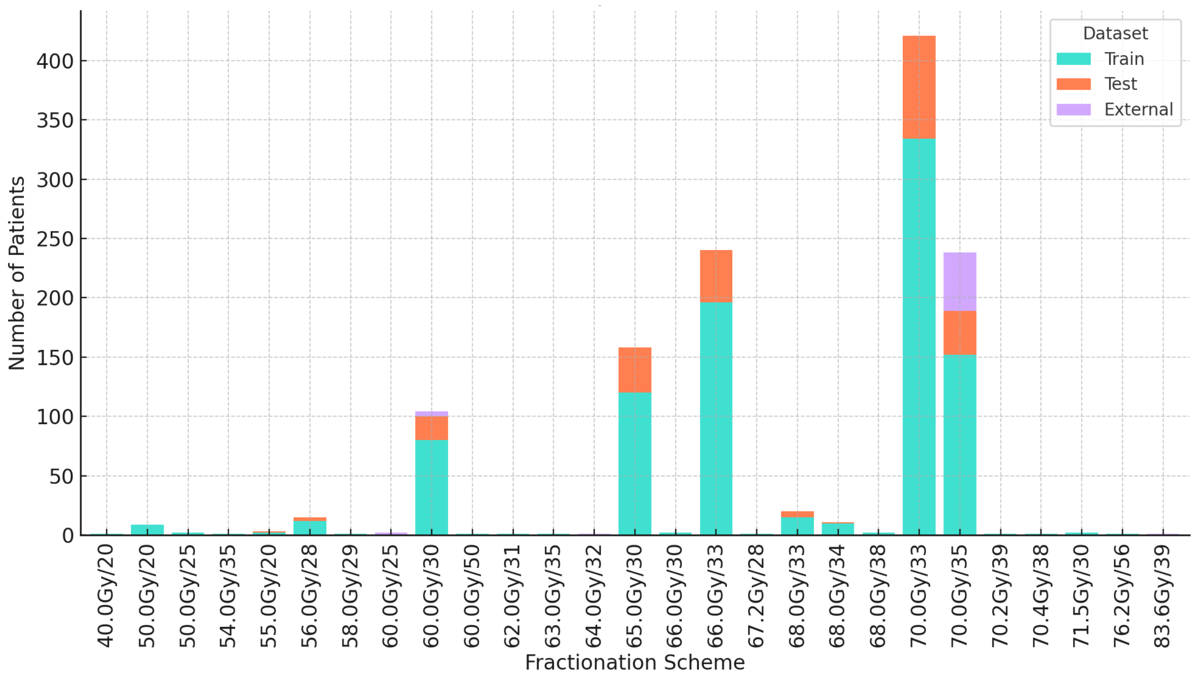

Figure E4. Patient distribution per fractionation scheme for the train, test and external datasets across institutions A to G (data from institution H was not directly available for aggregation in this plot).

Supplement F. Time to ORN analyses

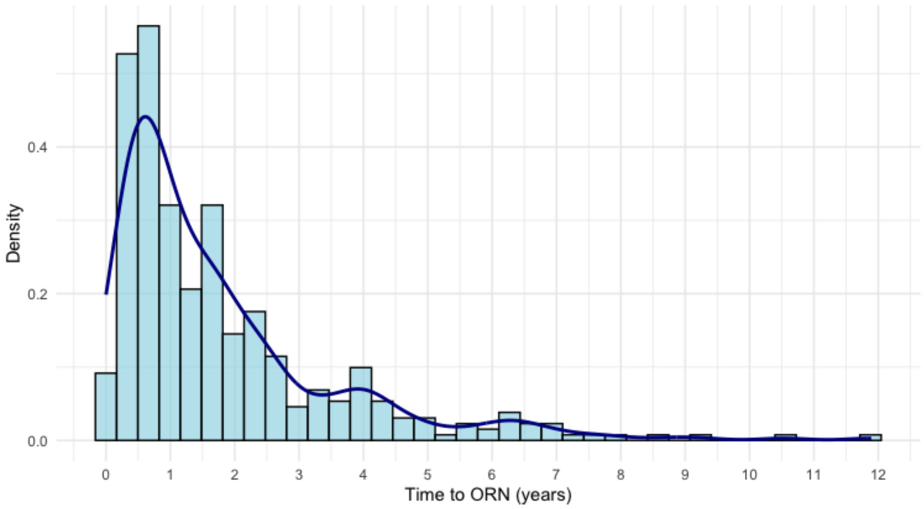

Figure F1. Distribution of time to ORN across institutions A to G (data from institution H was not directly available for aggregation in this plot). A density curve is overlaid to visualise the overall shape and concentration of the data, indicating the probability distribution of time to ORN.

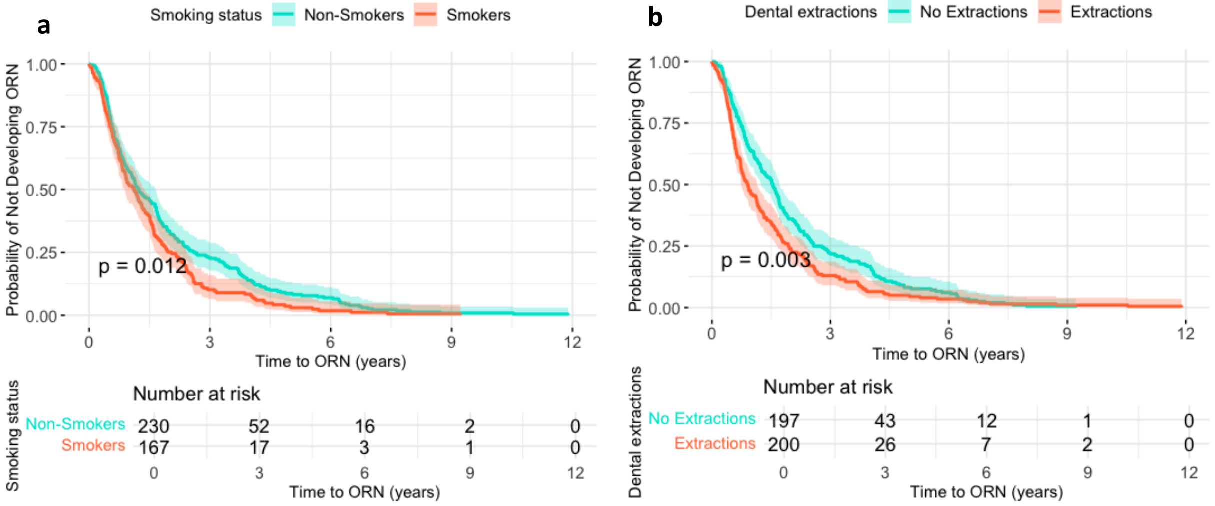

Figure F2. Kaplan-Meier survival curves across institutions A to G (data from institution H was not directly available for aggregation in this plot). Only patients with ORN are included and survival corresponds to ORN-free time after RT, stratified by sub-groups of ORN cases a) with and without pre-RT dental extractions and b) with positive (current) and negative (previous or never) smoking status. The shaded areas around each curve represent the 95% confidence intervals for the estimated survival probabilities over time. The statistical significance of the difference between the survival curves of the two groups was tested using the log-rank test, with a significance level of 0.05. The risk table below the plot displays the number of patients at risk in each group at various time points.

Supplement G. Multivariable stepwise forward logistic regression analyses

The Akaike Information Criterion (AIC) and the Likelihood Ratio Test (LRT) were used as the selection criteria with a threshold p-value of 0.05 for the latter. The AIC provides a means for model comparison by balancing a model's goodness of fit with model complexity (number of parameters used in the model), i.e., model parsimony. The LRT compares the goodness of fit between two nested models: a simpler model and a more complex model (i.e., simpler model with an additional variable in the forward stepwise approach).

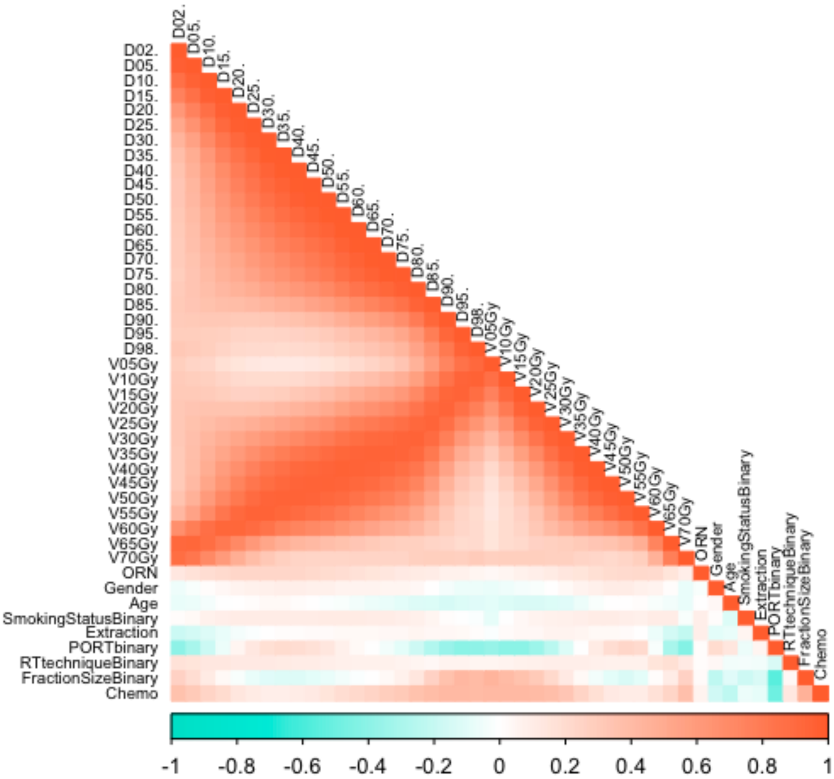

Figure G1. Spearman correlation matrix heatmap.

Table G1: Pre-selected variables and corresponding univariable p-values for the full cohort (i.e., including all primary tumour sites).

Variable	p-value	OR (95% CI)
$D_{30\%}$	<0.0001	1.06 (1.04, 1.08)
$D_{10\%}$	<0.0001	1.07 (1.04, 1.09)
$D_{60\%}$	<0.0001	1.03 (1.02, 1.04)
$V_{70\ Gy}$	<0.0001	1.05 (1.03, 1.07)
$V_{25\ Gy}$	0.0001	1.02 (1.01, 1.03)
Pre-RT dental extraction	0.003	1.51 (1.15, 1.98)
Smoking status	0.004	1.52 (1.15, 2.01)
$D_{95\%}$	0.006	1.02 (1.01, 1.04)
Chemotherapy	0.044	1.35 (1.01, 1.80)
Fraction size	0.412	1.12 (0.85, 1.47)
Sex	0.434	0.88 (0.64, 1.21)
RT technique	0.677	1.07 (0.78, 1.47)
PORT	0.722	1.06 (0.78, 1.43)
Age	0.977	1.00 (0.99, 1.01)

Table G2: Models evaluated during the stepwise forward selection process (all primary tumour sites). The thick line box indicates the model that was selected for this cohort.

Step	Variables	β	OR	p_val	AIC	BIC	p_LRT
1	Intercept	-3.849	0.02		1144.0	1153.7	<0.0001
	$D_{30\%}$	0.058	1.06	<0.0001			
2	Intercept	-4.102	0.02		1136.7	1151.2	0.002
	$D_{30\%}$	0.059	1.06	<0.0001			
	Dental extraction	0.437	1.55	0.002			
3	Intercept	-3.728	0.02		1133.5	1152.9	0.023
	$D_{30\%}$	0.050	1.05	<0.0001			
	Extraction	0.492	1.64	0.001			
	V_{70Gy}	0.024	1.02	0.030			
4	Intercept	-3.749	0.02		1154.8	1154.8	0.026
	$D_{30\%}$	0.048	1.05	<0.0001			
	Dental extraction	0.481	1.62	0.001			
	V_{70Gy}	0.025	1.03	0.024			
	Smoking status	0.333	1.39	0.026			
5	Intercept	-4.029	0.02		1159.5	1159.5	0.140
	$D_{30\%}$	0.051	1.05	<0.0001			
	Dental extraction	0.499	1.65	0.001			
	V_{70Gy}	0.020	1.02	0.084			
	Smoking status	0.370	1.45	0.015			
	Fraction Size	0.231	1.26	0.140			

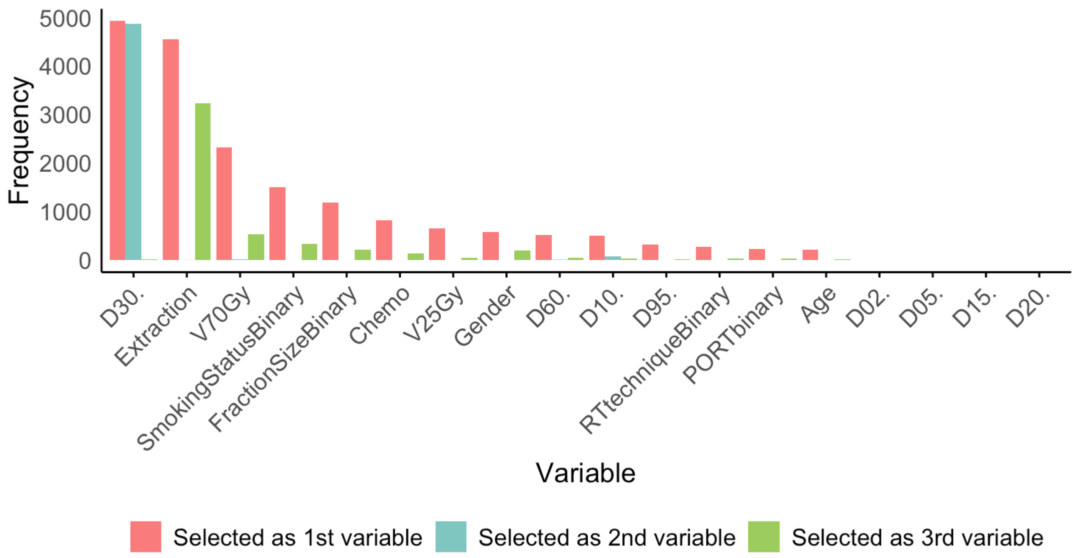

Figure G2: Bootstrapped variable selection frequency results.

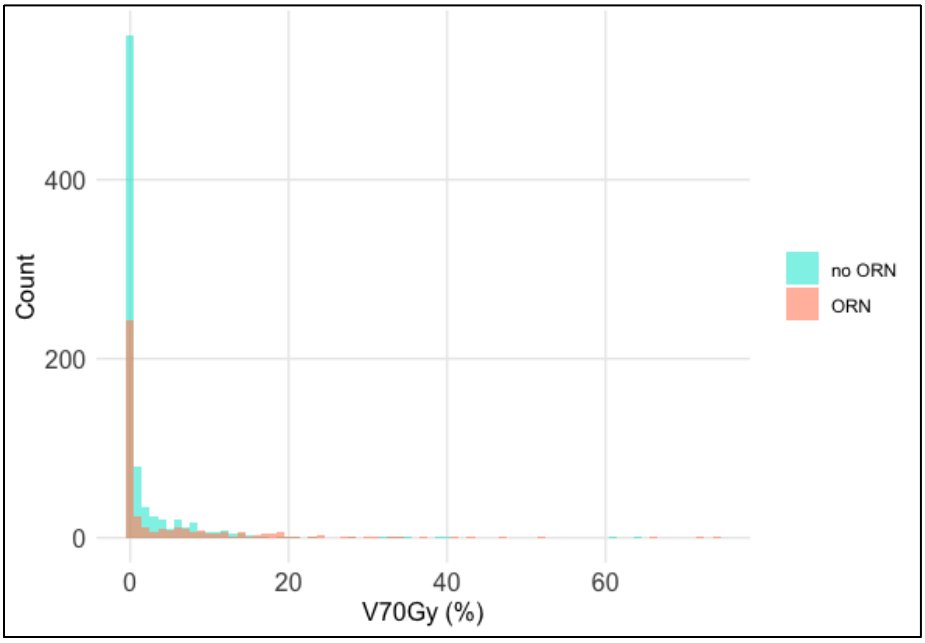

Figure G3. Distribution of the V_{70Gy} parameter stratified by osteoradionecrosis (ORN) status. The majority of patients received no high-dose exposure (V_{70Gy} = 0%), with a right-skewed tail representing small subsets of patients with higher values. Although a statistically significant difference was observed between groups, effect size was negligible (Cliff's delta = –0.13), suggesting limited clinical relevance.

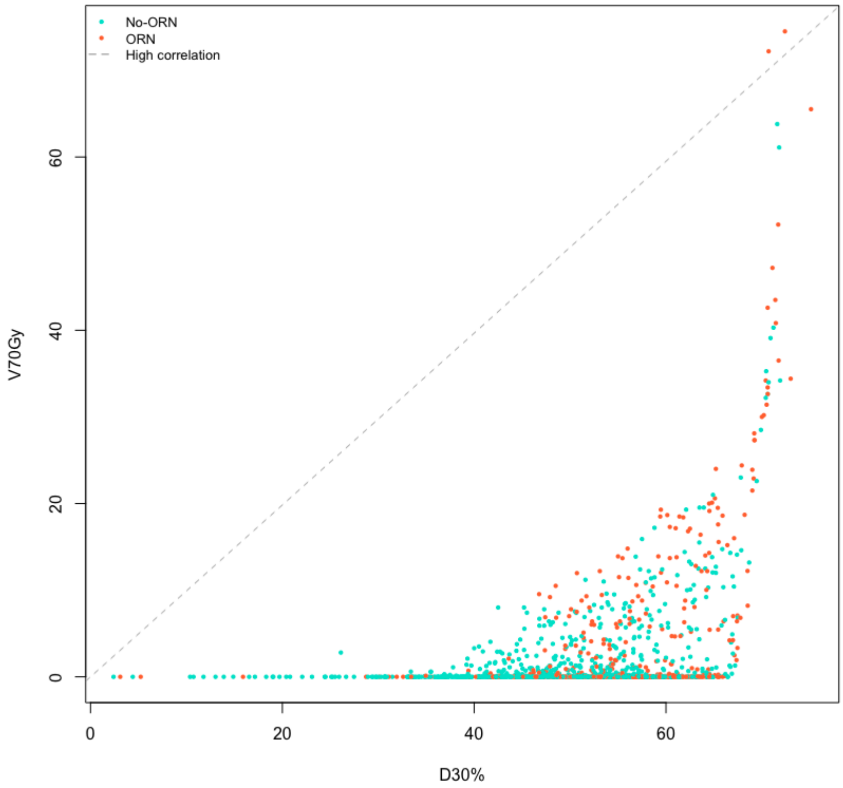

Figure G4. Scatter plot demonstrating the low collinearity between the two dosimetric variables selected in the final NTCP model, $D_{30\%}$ and V_{70Gy}.

Supplement H. Sub-cohort analyses

Table H1. Model performance results of the ORN NTCP model on the sub-cohorts of patients treated with OPC/advanced larynx/hypopharynx cancer or OCC. The confidence interval (CI) for the area under the ROC curve (AUC) has been calculated using the DeLong method.

**ORN NTCP model performance on the OPC/advanced larynx/hypopharynx cancer sub-cohort**			
	**Test (N=156)**	**External validation 1 (N=32)**	**External validation 2 (N=1054)**
ROC AUC (95% CI)	0.75 (0.67-0.83)	0.75 (0.58-0.92)	0.67 (0.62-0.73)
Nagelkerke R^2	0.253	0.255	0.062
Brier score	0.186	0.167	0.085
Log Loss	0.544	0.497	0.303
**ORN NTCP model performance on the OCC sub-cohort**			
	**Test (N=68)**	**External validation 1 (N=7)**	**External validation 2 (N=429)**
ROC AUC (95% CI)	0.53 (0.38-0.68)	n/a*	0.53 (0.45-0.60)
Nagelkerke R^2	0.008		0.001
Brier score	0.212		0.135
Log Loss	0.615		0.441

* External validation of the model on the OCC sub-cohort was not attempted due to the small sample sizes for this data subset (N=7).

Table H2. Model performance results of the ORN NTCP model on the sub-cohorts of patients treated with Primary RT or PORT. The confidence interval (CI) for the area under the ROC curve (AUC) has been calculated using the DeLong method.

**ORN NTCP model performance on the Primary RT sub-cohort**			
	**Test (N=176)**	**External validation 1 (N=54)**	**External validation 2 (N=1509)**
ROC AUC (95% CI)	0.70 (0.62-0.79)	0.72 (0.58-0.86)	0.67 (0.62-0.71)
Nagelkerke R^2	0.129	0.209	0.054
Brier score	0.170	0.190	0.077
Log Loss	0.512	0.555	0.283
**ORN NTCP model performance on the PORT sub-cohort**			
	**Test (N=60)**	**External validation 1 (N=4)**	**External validation 2 (N=429)**
ROC AUC (95% CI)	0.60 (0.45-0.74)	n/a*	0.52 (0.45-0.60)
Nagelkerke R^2	0.065		0.002
Brier score	0.210		0.135
Log Loss	0.606		0.441

* External validation of the model on the PORT sub-cohort was not attempted due to the small sample sizes for this data subset (N=4).

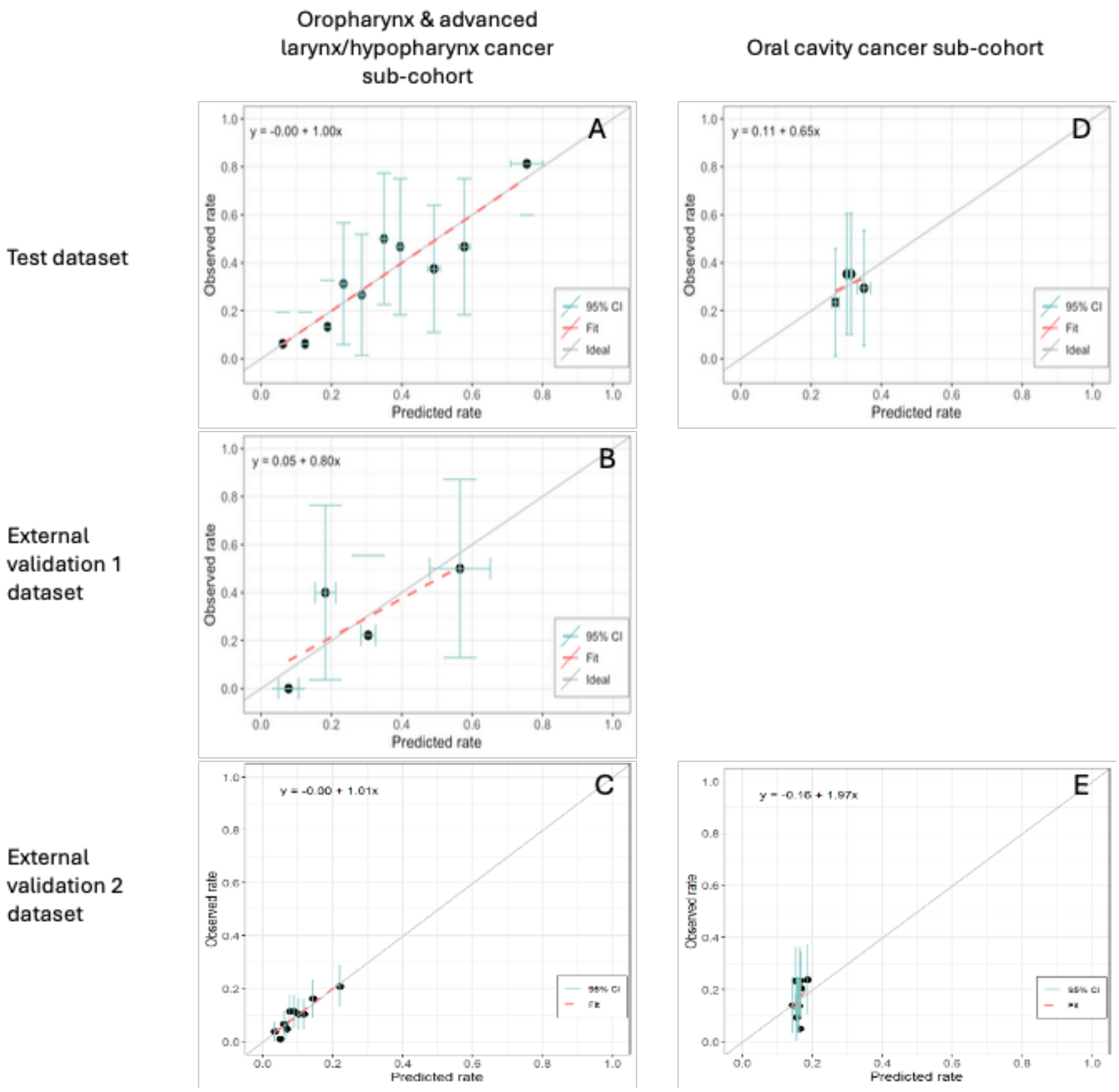

Figure H: Calibration curves when testing the NTCP model on the test and external validation datasets of the oropharynx (OPC)/advanced larynx/hypopharynx cancer (A, B, C) and the oral cavity cancer (OCC) (D, E) sub-cohorts. The number of bins in the calibration plots was adjusted in each plot based on dataset size for each cohort/sub-cohort to optimise representation of the data.

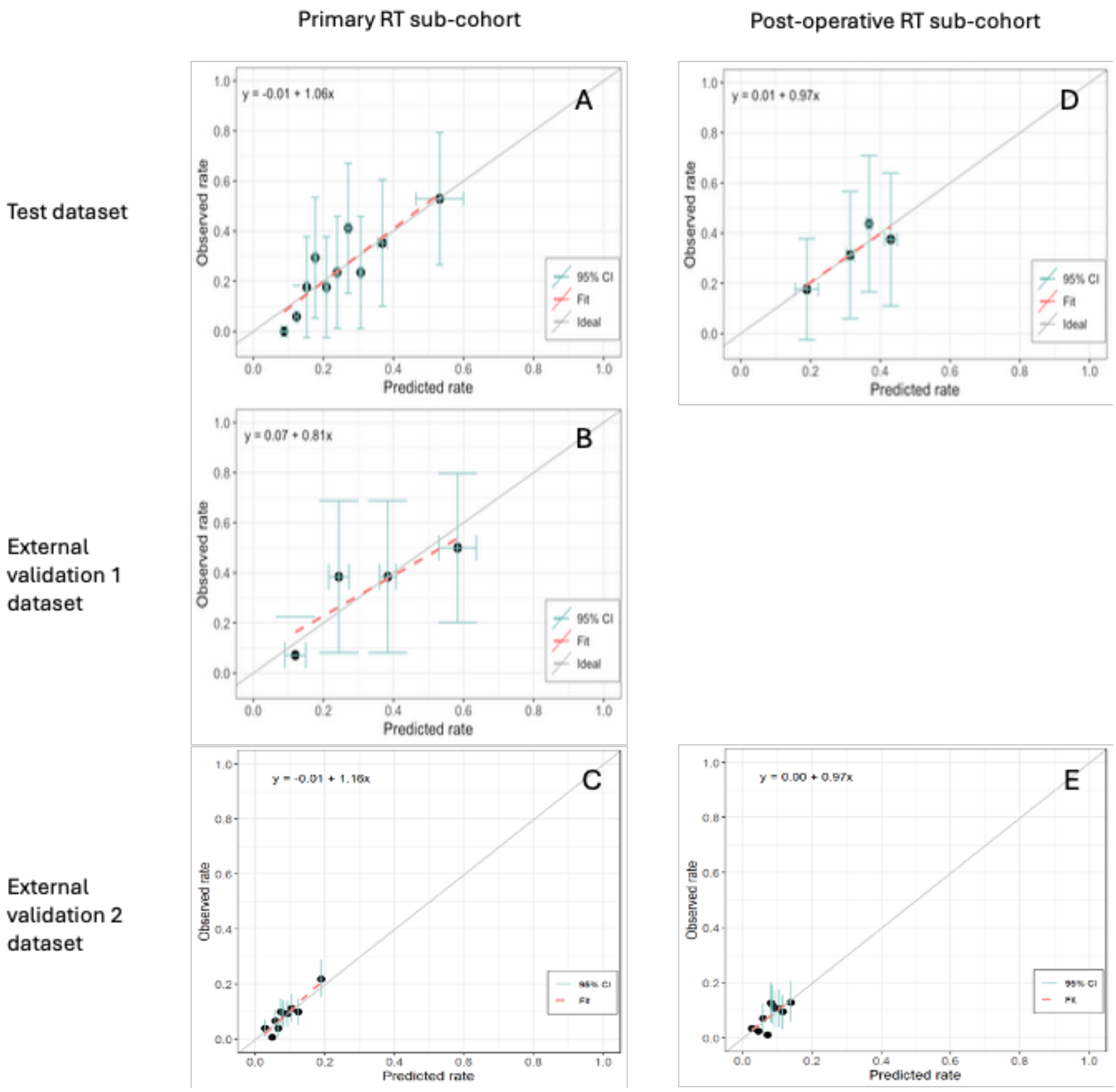

Figure H2: Calibration curves when testing the NTCP model on the test and external validation datasets of the Primary RT (A, B, C) and the post-operative RT (PORT) (D, E) sub-cohorts. The number of bins in the calibration plots was adjusted in each plot based on dataset size for each cohort/sub-cohort to optimise representation of the data.

Supplement I. EQD2 approximation

EQD2 approximation methodology: For D30%, we directly applied the linear–quadratic (LQ) model: $D30_{EQD2} = D30 \times \frac{d+{}^{\alpha}/_{\beta}}{2+{}^{\alpha}/_{\beta}}$, where *d* is the prescribed fraction size and α/β was set to 0.85 Gy for mandibular bone. For volume thresholds such as V70Gy, direct scaling is not possible without access to the raw DVH data since the threshold operation is non-linear. Instead, we approximated V70EQD2 by first calculating the EQD2 scaling factor *k*, then obtaining the corresponding physical threshold *Tphys* and then we estimate V70EQD2 as the DVH volume at *Tphys* using linear interpolation between the nearest available dose bins in our dataset (e.g., if d=2 Gy, then k=1 and *Tphys*=70 Gy, so the metric remains unchanged).

$$k = \frac{d + {}^{\alpha}/_{\beta}}{2 + {}^{\alpha}/_{\beta}}$$

$$Tphys = {}^{70}/_{k} = 70 \times \frac{2 + {}^{\alpha}/_{\beta}}{d + {}^{\alpha}/_{\beta}}$$

The performance comparison between models using physical dose and EQD2 approximation based D30% and V70Gy is in the tables and plots below. Note that, as already highlighted in the manuscript, we did not have direct access to the dataset used for the second external validation and therefore comparison has been done for training, testing and the first external validation.

Table I1. Original (physical dose) results.

	**Training (N=948)**	**Test (N=236)**	**External validation 1 (N=58)**	**External validation 2 (N=2687)**
ROC AUC	0.67	0.69	0.69	0.65
(95% CI)	(0.64-0.71)	(0.62-0.76)	(0.53-0.84)	(0.61-0.69)
Nagelkerke R^2	0.108	0.122	0.098	0.046
Brier score	0.203	0.204	0.199	0.077
Log Loss	0.591	0.594	0.596	0.281

Table I2. EQD2 approximation results.

	**Training (N=948)**	**Test (N=236)**	**External validation 1 (N=58)**
ROC AUC	0.68	0.69	0.68
(95% CI)	(0.64-0.71)	(0.62-0.76)	(0.52-0.83)
Nagelkerke R2	0.115	0.125	0.092
Brier score	0.201	0.203	0.201
Log Loss	0.588	0.593	0.598

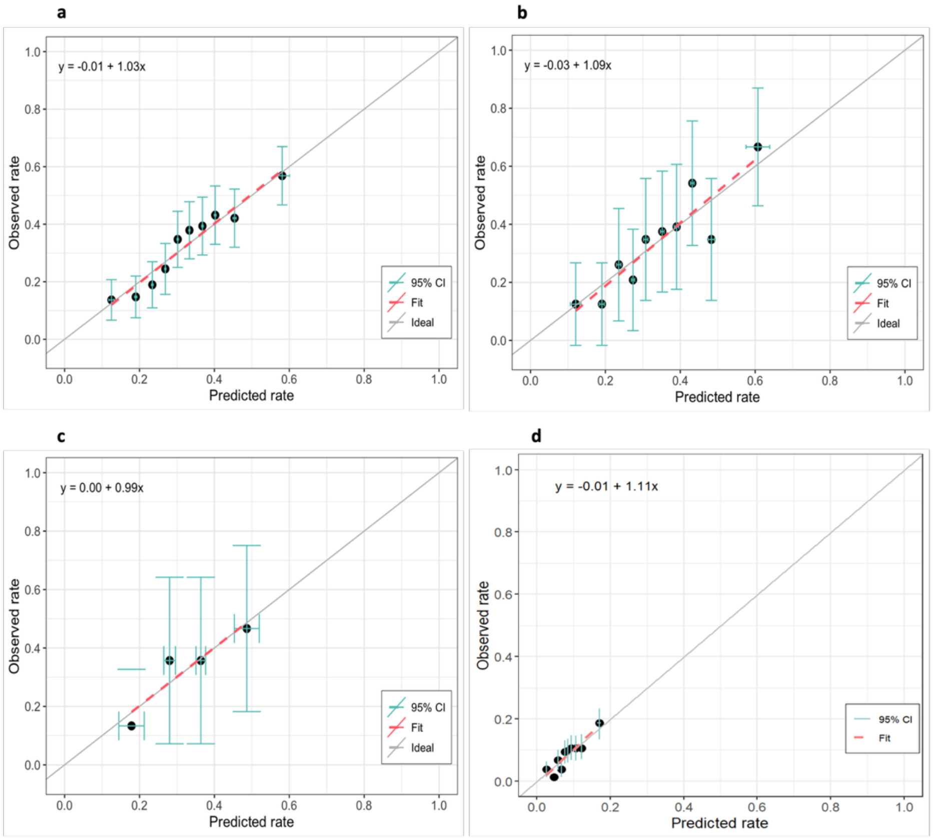

Figure I1. Original (physical dose) calibration curves for a) training, b) testing, c) first external validation and d) second external validation.

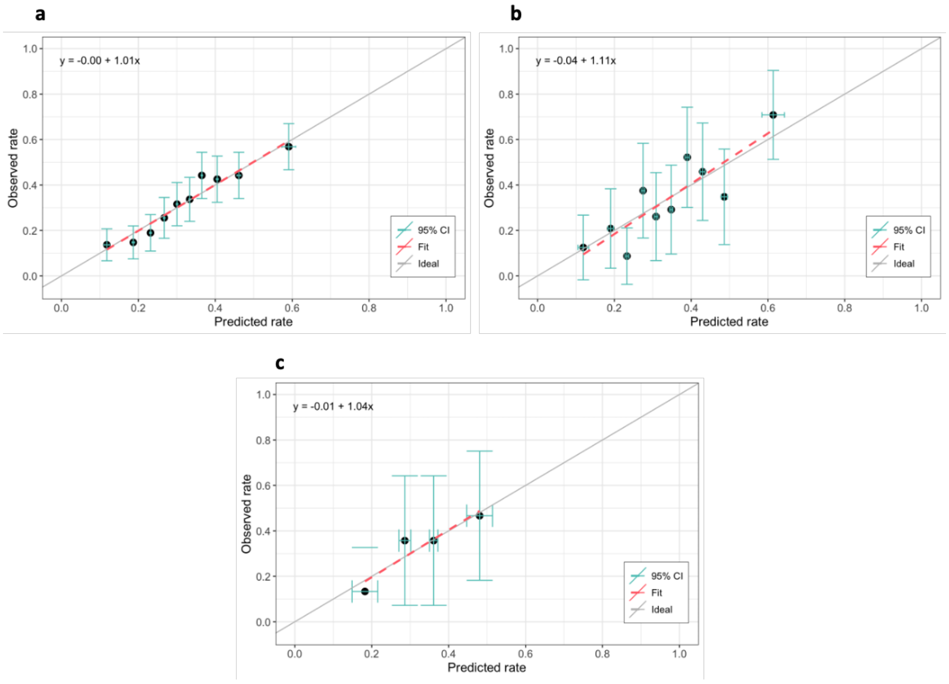

Figure I2. EQD2 approximation calibration curves for a) training, b) testing and c) first external validation. Data from institution H was not directly available for this analysis.

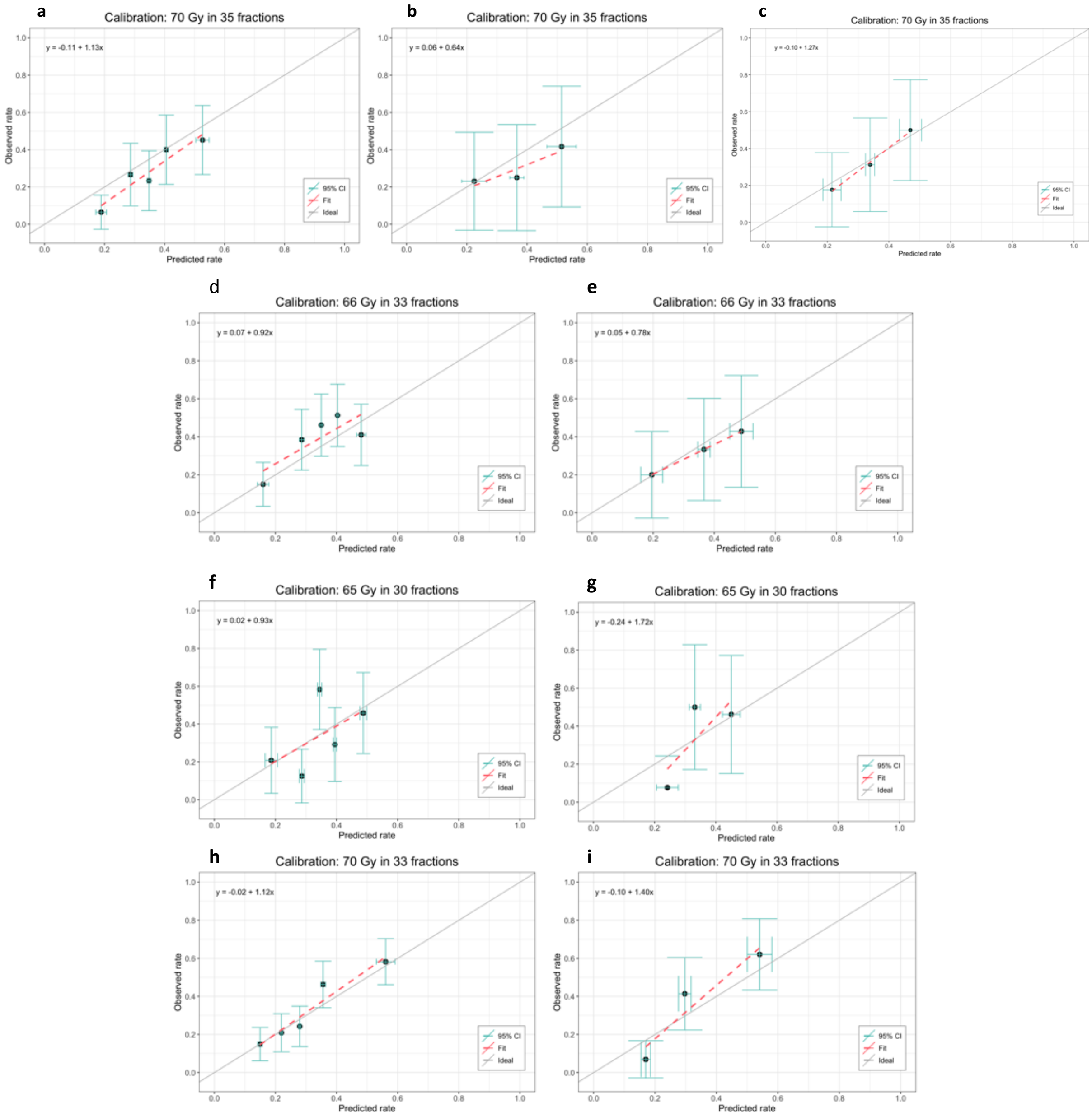

Figure I3. Calibration curves for subsets of patients treated with 70 Gy in 35 fractions [a) training, b) testing and c) external validation 1], 66 Gy in 33 fractions [d) training and e) testing], 65 Gy in 30 fractions [f) training and g) testing], and 70 Gy in 33 fractions [h) training and i) testing]. Data from institution H was not directly available for this analysis. Calibrations curves for external validation were limited by small patient numbers in fractionation schemes other than 70 Gy in 35 fractions (see details in Figure E4).

Supplement J. TRIPOD checklist

Section/Topic			Checklist Item	Page
**Title and abstract**				
Title	1	D;V	Identify the study as developing and/or validating a multivariable prediction model, the target population, and the outcome to be predicted.	1
Abstract	2	D;V	Provide a summary of objectives, study design, setting, participants, sample size, predictors, outcome, statistical analysis, results, and conclusions.	2
**Introduction**				
Background and objectives	3a	D;V	Explain the medical context (including whether diagnostic or prognostic) and rationale for developing or validating the multivariable prediction model, including references to existing models.	3
	3b	D;V	Specify the objectives, including whether the study describes the development or validation of the model or both.	4
**Methods**				
Source of data	4a	D;V	Describe the study design or source of data (e.g., randomized trial, cohort, or registry data), separately for the development and validation data sets, if applicable.	4
	4b	D;V	Specify the key study dates, including start of accrual; end of accrual; and, if applicable, end of follow-up.	4
Participants	5a	D;V	Specify key elements of the study setting (e.g., primary care, secondary care, general population) including number and location of centres.	4
	5b	D;V	Describe eligibility criteria for participants.	4/Sup. A
	5c	D;V	Give details of treatments received, if relevant.	11/Sup. B
Outcome	6a	D;V	Clearly define the outcome that is predicted by the prediction model, including how and when assessed.	6/Sup. B
	6b	D;V	Report any actions to blind assessment of the outcome to be predicted.	
Predictors	7a	D;V	Clearly define all predictors used in developing or validating the multivariable prediction model, including how and when they were measured.	5/Sup. A, B
	7b	D;V	Report any actions to blind assessment of predictors for the outcome and other predictors.	
Sample size	8	D;V	Explain how the study size was arrived at.	5
Missing data	9	D;V	Describe how missing data were handled (e.g., complete-case analysis, single imputation, multiple imputation) with details of any imputation method.	8
Statistical analysis methods	10a	D	Describe how predictors were handled in the analyses.	5
	10b	D	Specify type of model, all model-building procedures (including any predictor selection), and method for internal validation.	6/7
	10c	V	For validation, describe how the predictions were calculated.	6/7
	10d	D;V	Specify all measures used to assess model performance and, if relevant, to compare multiple models.	6/7
	10e	V	Describe any model updating (e.g., recalibration) arising from the validation, if done.	Sup. I
Risk groups	11	D;V	Provide details on how risk groups were created, if done.	
Development vs. validation	12	V	For validation, identify any differences from the development data in setting, eligibility criteria, outcome, and predictors.	Sup. B, D, E
**Results**				
Participants	13a	D;V	Describe the flow of participants through the study, including the number of participants with and without the outcome and, if applicable, a summary of the follow-up time. A diagram may be helpful.	7/8
	13b	D;V	Describe the characteristics of the participants (basic demographics, clinical features, available predictors), including the number of participants with missing data for predictors and outcome.	11/Sup. B, D, E
	13c	V	For validation, show a comparison with the development data of the distribution of important variables (demographics, predictors and outcome).	Sup. B, D, E
Model development	14a	D	Specify the number of participants and outcome events in each analysis.	Sup. B, D, E
	14b	D	If done, report the unadjusted association between each candidate predictor and outcome.	8/Sup. C
Model specification	15a	D	Present the full prediction model to allow predictions for individuals (i.e., all regression coefficients, and model intercept or baseline survival at a given time point).	13/Sup. I
	15b	D	Explain how to the use the prediction model.	Sup. I
Model performance	16	D;V	Report performance measures (with CIs) for the prediction model.	14/Sup. G, H
Model-updating	17	V	If done, report the results from any model updating (i.e., model specification, model performance).	
**Discussion**				
Limitations	18	D;V	Discuss any limitations of the study (such as nonrepresentative sample, few events per predictor, missing data).	15-19
Interpretation	19a	V	For validation, discuss the results with reference to performance in the development data, and any other validation data.	15-19
	19b	D;V	Give an overall interpretation of the results, considering objectives, limitations, results from similar studies, and other relevant evidence.	15-19
Implications	20	D;V	Discuss the potential clinical use of the model and implications for future research.	15-19
**Other information**				
Supplementary information	21	D;V	Provide information about the availability of supplementary resources, such as study protocol, Web calculator, and data sets.	Supplementary material
Funding	22	D;V	Give the source of funding and the role of the funders for the present study.	13
